# Extreme-Climate-Driven Agricultural Trade Risk Sensing with Multimodal Consistency Learning and Edge Intelligence

**DOI:** 10.3390/s26196125

**Published:** 2026-09-27

**Authors:** Zijian Zhou, Ruijia Liu, Xiangchen Long, Yongbiao Hu, Fei Xia, Xi He, Yihong Song

**Affiliations:** 1 Peking University, Beijing 100871, China; 2 China Agricultural University, Beijing 100084, China

**Keywords:** extreme climate, agricultural risk, multimodal sensing, logistics risk prediction, uncertainty estimation

## Abstract

Extreme climate events increasingly amplify agricultural trade security risks across interconnected stages: agricultural production, commodity conditions, cold-chain storage, logistics distribution, and trade fulfillment. However, existing approaches predominantly focus on isolated stages and fail to model cross-stage risk propagation, enable real-time edge inference, or provide well-calibrated risk warnings. To address these challenges, a multimodal edge-intelligence framework, termed AgriClimate-EdgeNet, is proposed to jointly model climatic conditions, agricultural production, commodity imagery, cold-chain states, logistics trajectories, and trade records. An extreme-climate-aware cross-modal consistency mechanism is developed to capture normal inter-stage correspondence and identify abnormal information conflicts. Depthwise separable temporal convolutions, gated temporal units, lightweight attention, and Teacher–Student distillation are incorporated for efficient edge inference. Furthermore, dynamic modality reliability estimation and dual predictive uncertainty modeling (decoupling epistemic and heteroscedastic aleatoric uncertainties) are integrated to ensure decision trustworthiness under severe sensory noise and missing observations. On a 38,400-window agricultural trade dataset, AgriClimate-EdgeNet achieves an Accuracy of 0.914, Recall of 0.896, Macro-F1 of 0.902, and AUC of 0.949, while reducing expected calibration error to 0.028 in routine single-pass Streaming Mode and 0.021 under multi-sample Deep Audit Mode. In operational edge deployment on NVIDIA Jetson AGX Orin, the model requires 3.96 M on-device parameters and 1.21 G FLOPs, achieving an inference latency of 8.6 ms (116.3 samples/s) in Streaming Mode and 38.4 ms in Deep Audit Mode. These results demonstrate that AgriClimate-EdgeNet provides an accurate, robust, and low-latency solution for full-chain agricultural trade risk sensing under extreme climate shocks.

## 1. Introduction

Agricultural trade is a multistage system encompassing production, harvesting, storage, cold-chain transportation, logistics, port clearance, and market transactions, whose stability is critical to producer profitability, agricultural supply security, and interregional trade resilience [1]. Increasingly frequent extreme climate events, such as heatwaves, droughts, floods, and typhoons, can propagate beyond agricultural production along the production–commodity–storage–logistics–trade chain [2]. For example, high temperatures can simultaneously reduce crop yield and quality and increase cold-chain refrigeration loads, while floods can disrupt transportation networks and aggravate port congestion, ultimately causing commodity losses, delivery delays, and trade risks [3]. Agricultural trade security under extreme climate conditions should therefore be formulated as a multisource, cross-stage, dynamic risk propagation and early-warning problem rather than an isolated meteorological forecasting or trade anomaly detection task [4]. Meanwhile, agricultural Internet of things (IoT), cold-chain sensors, global positioning system (GPS)/automatic identification system (AIS), unmanned aerial vehicles (UAVs), intelligent warehousing, and digital trade platforms provide multimodal observations of climate, production, commodities, transportation, and trade processes, creating the data foundation for tracing downstream risks from upstream production [5].

In conceptualizing this risk propagation mechanism, the pathway of Extreme Climate → Production → Commodity → Cold Chain → Logistics → Trade represents a sequential, physically and administratively coupled transmission process rather than simple hierarchical inclusion relationships. Specifically, “Production” governs upstream agronomic growth, soil dynamics, and field crop health under open-air meteorological stress; “Commodity” characterizes postharvest biological qualities, including physiological firmness, moisture retention, surface defects, and natural perishability; “Cold Chain” represents thermodynamic microclimate regulation across precooling stations, refrigerated warehouses, and reefer compartments; “Logistics” encompasses macro spatiotemporal fleet movements, GPS/AIS transit trajectories, road accessibility, and intermodal transshipment; and “Trade” reflects commercial, financial, and legal fulfillment, including contracts, phytosanitary clearance, customs declarations, and delivery acceptance. Although these stages are operationally coordinated, conflating them into coarse inclusion categories (such as subsuming commodity into trade, or cold chain into logistics) masks the true underlying failure mechanisms. For example, a refrigerated truck may progress according to schedule along its planned logistics trajectory (normal logistics), yet an external heatwave may overload its refrigeration compressor, causing persistent temperature excursions (cold-chain anomaly) that accelerate physiological softening and rot (commodity deterioration), which only culminates in shipment rejection, price deductions, or legal disputes (trade disruption) upon arrival inspection days later. Disentangling and modeling this continuous propagation pathway is therefore essential for preemptive, stage-traceable risk warning.

Existing agricultural and trade risk studies mainly employ statistical, time-series, and machine-learning methods such as Random Forest and XGBoost using meteorological, production, price, logistics, or trade data [6]. However, most approaches model individual stages independently [7]: climate-risk studies primarily examine impacts on crop yield, quality, and disaster probability, whereas supply-chain and trade studies mainly rely on downstream logistics and transaction records, with limited integration of upstream climate and production states [8]. Critically, existing domain-specific agricultural climate models (such as crop-yield and drought forecasting models) terminate at the farm gate, treating agricultural harvest as the final endpoint and failing to account for postharvest thermodynamic stress or transport network vulnerabilities. Conversely, conventional trade anomaly and supply chain models operate strictly on downstream tabular invoices, customs logs, and order records, remaining blind to upstream meteorological forcing until delays or contract defaults have physically materialized. Furthermore, while generic multimodal deep learning architectures have emerged, they commonly presume uncorrupted sensor feeds and cooperative intermodal consensus. In practical agricultural trade under extreme weather, existing approaches exhibit two fundamental deficiencies: first, they lack explicit cross-modal conflict identification mechanisms to recognize anomalies when declared documents contradict physical sensor observations (such as “on-schedule” trade paperwork paired with stalled GPS coordinates or elevated reefer temperatures); second, they lack robustness under extreme environmental degradation, where severe storms or heatwaves induce widespread sensor drift, telemetry dropouts, and communication loss, often leading conventional architectures to output disastrous high-confidence erroneous predictions [9,10]. Moreover, meteorological observations, field sensing signals, commodity imagery, cold-chain measurements, GPS trajectories, and trade events differ substantially in sampling frequency, temporal scale, and data structure. Consequently, conventional stage-specific modeling and simple feature concatenation cannot adequately characterize the continuous propagation pathway of Extreme Climate → Production → Commodity → Cold Chain → Logistics → Trade, motivating integrated multimodal modeling of cross-stage agricultural trade risks [11].

Recent advances in long short-term memory (LSTM), gated recurrent unit (GRU), temporal convolutional network (TCN), Transformer, and multimodal deep learning approaches have enabled nonlinear temporal modeling and joint representation of heterogeneous agricultural information, including climate, production, imagery, cold-chain, logistics, and trade data [12,13]. Multimodal sensing further demonstrates that heterogeneous signals with different data structures and sampling characteristics can complement each other in complex state recognition [14]. However, existing methods primarily optimize final predictive performance without explicitly distinguishing normal cross-modal correspondence from abnormal information conflicts [9]. For example, normal trade or logistics records may coexist with GPS stagnation or climate-induced cold-chain anomalies [10]. Such inconsistencies may constitute early signals of climate-induced risks before they evolve into explicit trade disruptions, making cross-modal inconsistency modeling essential for early warning [15]. Agricultural trade risks often emerge at distributed nodes such as farms, warehouses, refrigerated vehicles, and ports, requiring low-latency inference close to data sources [16]. Compared with centralized cloud processing, edge computing can reduce communication latency and transmission overhead, while lightweight techniques such as structural compression, depthwise separable convolution, pruning, quantization, and knowledge distillation make deployment under constrained computational resources feasible [17,18]. Nevertheless, extreme climate conditions can cause sensor noise, GPS drift, missing data, and communication interruptions, requiring edge models to preserve not only predictive accuracy but also robustness, reliability, and uncertainty awareness [19,20]. Recent studies provide supporting evidence for this direction: AMAN demonstrates multimodal climate-aware prediction and cross-regional adaptation [21]; Farm-LightSeek enables compressed multimodal agricultural intelligence on edge devices [22]; FEL-MSF achieves communication-efficient, low-latency federated multimodal sensing [23]; and edge-oriented multimodal remote-sensing fusion supports efficient processing of heterogeneous environmental observations [24]. These advances motivate a unified framework that integrates multimodal sensing, cross-modal inconsistency modeling, lightweight edge inference, reliability estimation, and dual (epistemic and heteroscedastic aleatoric) uncertainty quantification for real-time agricultural trade risk warning under extreme climate conditions [25].

To address the aforementioned limitations, a multimodal edge-intelligence framework for agricultural trade security under extreme climate conditions, termed AgriClimate-EdgeNet, is proposed. A multimodal sensing architecture covering climatic environments, agricultural production, commodity imagery, storage and cold-chain conditions, logistics transportation, and trade information is first established, while asynchronous and multi-frequency observations are associated through a unified temporal window and trade-event linkage mechanism. Extreme-climate-aware cross-modal consistency modeling is subsequently employed to learn relationships among climate–production, production–trade, visual–document, cold chain–commodity, and logistics–document modalities, thereby identifying abnormal information conflicts induced by climate shocks. A lightweight edge prediction network based on depthwise separable temporal convolution, gated temporal structures, and lightweight attention is then constructed, while teacher–student knowledge distillation is incorporated to reduce model parameters, floating-point operations (FLOPs), memory consumption, and inference latency. Finally, modality reliability is dynamically estimated according to data missing rates, sensor noise, sampling delays, and signal stability, and dual uncertainty modeling is further incorporated to jointly output risk probabilities, risk levels, epistemic and aleatoric uncertainty, and major risk sources. The main contributions of this study are summarized as follows.

1. A unified multimodal sensing and risk propagation architecture is established across the entire agricultural trade chain (Extreme Climate → Production → Commodity → Cold Chain → Logistics → Trade), transforming isolated post-hoc trade prediction into full-process, real-time proactive risk sensing.
2. An extreme-climate-aware cross-modal consistency modeling mechanism is proposed. Normal correspondence relationships among different stages of agricultural trade are explicitly learned through cross-modal attention, contrastive learning, and consistency matching, while cross-modal conflict representations are used to identify abnormal propagation processes induced by climate shocks. Consequently, the model is capable not only of determining whether a risk exists but also of characterizing the cross-stage evidence underlying its formation.
3. A lightweight temporal risk prediction network suitable for edge deployment is designed. Through lightweight temporal modeling and teacher–student knowledge distillation, parameters, FLOPs, memory, and inference latency are reduced while predictive performance is preserved as much as possible. The resulting model can therefore be deployed on edge nodes located at farms, warehouses, cold-chain vehicles, logistics facilities, and ports to support low-latency risk warning.
4. Extreme-climate-driven data augmentation, modality reliability estimation, and dual predictive uncertainty modeling (disentangling epistemic model uncertainty from heteroscedastic aleatoric sensory noise) are formulated. The contribution of each modality is dynamically recalibrated under sensor degradation, missing observations, and severe climate disruptions, suppressing untrustworthy signals and elevating predictive uncertainty to prevent erroneous high-confidence decisions.

## 2. Related Work

### 2.1. Agricultural Trade and Supply Chain Risk Analysis

Agricultural trade and supply chain risk analysis aims to identify evolving risk factors across production, storage, transportation, transaction, and fulfillment processes and predict supply disruptions, price anomalies, logistics delays, defaults, and fraud through risk classification, forecasting, and anomaly detection [26]. Early studies mainly employed regression, time-series analysis, risk scoring, and multi-indicator evaluation based on commodity prices, transaction volumes, inventories, enterprise credit, and fulfillment records [27]. Although interpretable, these methods rely heavily on predefined statistical relationships and are therefore limited in modeling nonlinear, time-varying, and interacting risks [28]. With the digitalization of agricultural supply chains, Random Forest and XGBoost have been introduced to learn nonlinear relationships from multidimensional transaction, logistics, and operational data [29,30]. However, their reliance on static feature representations limits the modeling of temporally accumulated risks, particularly those induced by extreme climate shocks [31]. Temporal models such as LSTM, GRU, TCN, and Transformer further capture long-term dependencies for predicting price fluctuations, demand changes, logistics delays, and supply chain anomalies [32]. Trade documents have also been combined with GPS/AIS trajectories to detect inconsistencies between declared transactions and physical cargo movements [33]. In parallel, an expanding body of research has investigated the multifaceted vulnerabilities of agricultural supply chains to extreme meteorological shocks across distinct operational stages. Ambient extreme temperatures accelerate postharvest respiration and tissue decay in perishable fruits and vegetables, while simultaneously driving up refrigeration power requirements and increasing the incidence of cold-chain equipment failures [34]. Extreme precipitation events, flash floods, and coastal typhoons cause severe physical washouts of rural transportation infrastructure and precipitate widespread maritime demurrage and terminal congestion at commercial trade ports [35]. Furthermore, compound climatic extremes systematically destabilize interregional food commodity availability, leading to severe supply disruptions and price volatility across global value networks [36,37].

In summary, previous research has established critical empirical foundations for understanding agricultural supply chain risks, but three key limitations remain evident: (1) existing models predominantly operate in stage-isolated silos, treating agronomic production, postharvest cold storage, freight transit, and commercial trading as independent problems; (2) upstream physical climate impacts and downstream economic trade outcomes are decoupled, precluding end-to-end tracking of risk cascading; and (3) conventional fusion approaches lack the capacity to identify and exploit cross-modal information conflicts when physical sensing streams contradict administrative trade records. These challenges highlight the need for an integrated, full-chain risk sensing paradigm capable of operating reliably under extreme weather shocks.

### 2.2. Climate-Aware Agricultural Sensing and Multimodal Modeling

Climate-aware agricultural modeling integrates environmental and agricultural observations to characterize the dynamic relationship among climate variation, crop response, and production status [38]. Meteorological stations and reanalysis datasets such as ERA5 provide large-scale temperature, humidity, precipitation, wind, and other meteorological fields for identifying extreme events [39], while agricultural IoT sensors complement them with localized measurements of field and soil conditions [40]. Remote sensing and UAV imagery further capture vegetation status, crop stress, and disaster impacts, whereas near-ground visual observations characterize commodity maturity, appearance, and quality [41,42]. After harvest, cold-chain sensors extend this sensing process to storage and transportation by continuously monitoring environmental conditions and detecting temperature excursions, refrigeration abnormalities, and potential quality deterioration [43]. Multimodal agricultural sensing therefore integrates meteorological, IoT, remote-sensing, visual, and cold-chain information across spatial, temporal, and operational stages to provide complementary representations of agricultural system states [44]. Existing climate-aware models have demonstrated the effects of extreme conditions on crop stress, yield, and quality, but generally treat agricultural production as the final prediction target. Consequently, how these upstream impacts subsequently increase cold-chain loads or evolve into transportation disruptions, port delays, and trade fulfillment risks remains insufficiently modeled [45]. This study therefore extends climate-aware multimodal sensing from production-oriented prediction toward full-process agricultural trade risk sensing, with particular emphasis on tracing the continuous propagation of extreme climate shocks from agricultural production to downstream cold-chain, logistics, and trade stages.

### 2.3. Edge Intelligence and Robust Risk Sensing

Edge intelligence performs data processing and model inference close to data sources, reducing the latency and bandwidth costs of continuously transmitting agricultural sensing data to the cloud [46]. In agricultural trade systems, deploying models on field gateways, cold-chain vehicles, warehouses, and port nodes enables rapid detection of heat stress, temperature anomalies, vehicle stagnation, and logistics delays. Because these devices have limited computational and storage resources, lightweight techniques such as depthwise separable convolution, pruning, quantization, and knowledge distillation are commonly employed. In particular, knowledge distillation transfers knowledge from a high-capacity cloud Teacher to a compact edge Student, reducing inference costs while retaining predictive performance [47]. However, low-latency inference alone does not ensure reliable risk sensing [48]. Extreme climate conditions can introduce sensor noise, communication interruptions, GPS drift, and missing modalities, requiring models to account for data reliability and predictive uncertainty [49]. MC Dropout and Deep Ensemble estimate uncertainty from stochastic predictions or model disagreement, while reliability-aware multimodal fusion adjusts modality contributions according to missingness, noise, delay, and signal stability, reducing the influence of degraded information sources [50,51]. Although these techniques have been applied to agricultural IoT, equipment fault detection, and industrial monitoring, existing studies mainly address isolated production or equipment systems [52]. A unified framework integrating climate, production, commodity, cold-chain, logistics, and trade information with lightweight, reliable, and uncertainty-aware edge inference remains underexplored. This gap motivates the development of AgriClimate-EdgeNet for real-time agricultural trade risk warning under extreme climate conditions.

## 3. Materials and Method

### 3.1. Data Collection

A multimodal agricultural trade dataset was constructed around the continuous risk propagation chain of “extreme climate–agricultural production–commodity status–cold chain–logistics–trade”. Field sensing, cold-chain telemetry, and logistics tracking were anchored across 8 concurrent regional export corridors connecting major fresh produce production clusters in North China—originating from vegetable greenhouse and field cultivation clusters in Shouguang (Shandong Province) and surrounding production zones, transiting through provincial cold-chain logistics hubs and sensor monitoring stations in Yongqing County (Langfang City, Hebei Province), and terminating at maritime container terminals in Tianjin Port, China—spanning May 2023 to October 2025, as summarized in Table 1. The monitored agricultural commodities encompass high-value perishables representative of interregional export corridors, specifically Solanaceae crops (fresh-market tomatoes, sweet peppers), Cucurbitaceae (cucumbers), Brassicaceae (Chinese cabbage), and seasonal stone fruits. From the raw continuous telemetry across these corridors, 39,820 candidate sliding observation windows (L=6h, step S=1h) were initially generated. Rigorous quality auditing eliminated 620 windows intersecting the 48-h purge buffers, 480 windows lacking complete 24-h lookahead ground-truth follow-up records, and 320 windows with severe sensor transmission dropouts (>30%), yielding exactly *N* = 38,400 fully validated, non-leaking observation windows. Across all 38,400 windows, ambient meteorological conditions comprise Normal (58.2%, 22,349 windows), Extreme Heat (14.8%, 5683 windows), Heavy Rainfall/Flooding (12.1%, 4646 windows), Severe Drought (9.4%, 3610 windows), and Gale/Strong Wind (5.5%, 2112 windows).

Climate and environmental data were obtained from ground-based meteorological stations, agricultural IoT monitoring nodes, and ERA5 reanalysis products. Variables including air temperature, relative humidity, precipitation, wind speed, solar radiation, atmospheric pressure, and soil moisture were continuously recorded. Derived indicators, including heatwave duration, extreme precipitation intensity, drought severity, and abnormal wind speed, were further calculated according to historical hourly distributions. Ground-based sensors operated continuously at minute-level sampling frequencies, whereas ERA5 variables were extracted at an hourly resolution and matched with agricultural production batches according to the spatial coordinates of the observation areas. Based on regional historical climate quantiles and meteorological disaster records, each observation window was further categorized as normal, extreme heat, drought, heavy rainfall, flooding, or strong wind, thereby characterizing environmental variations before, during, and after extreme climate events. Agricultural production and commodity-status data were primarily obtained from cooperative production bases and postharvest sorting processes. During sowing, growth, maturation, and harvesting stages, soil temperature and moisture, electrical conductivity, canopy environmental conditions, and manually recorded agricultural operations were continuously monitored, while crop growth status, harvest date, batch identifier, yield, and quality grade were simultaneously documented. Visual data were jointly acquired using fixed cameras, handheld imaging devices, and UAV platforms. Fixed cameras were employed to generate continuous crop-growth image sequences, whereas UAVs conducted low-altitude surveys along predefined flight routes during key growth stages and before and after extreme weather events. After agricultural products entered the postharvest stage, images of external appearance, maturity, mechanical damage, surface defects, and packaging conditions were captured from multiple viewing angles. In addition, internal biochemical quality parameters, soluble solids content (Brix), and tissue elasticity were non-destructively measured using Vis-NIR diffuse reflectance probes (Ocean Insight Maya2000 Pro, Orlando, FL, USA, spectral range 200–1100 nm, 18,200 spectral scans) to detect latent sub-epidermal physiological breakdown prior to superficial skin discoloration. All visual and spectral samples were associated with the corresponding climate and production records through acquisition timestamps, plot identifiers, or commodity batch numbers, enabling production-state variations to be continuously traced to postharvest commodity conditions.

Cold-chain and warehousing data were obtained from precooling facilities at production sites, regional storage nodes, and refrigerated transport vehicles. Temperature, humidity, refrigeration-system operating status, vehicle-compartment or warehouse-door opening events, and storage duration were continuously monitored. Batch-specific features, including temperature-excursion duration, temperature fluctuation amplitude, cumulative thermal exposure, and refrigeration recovery time, were subsequently derived. Sensor terminals recorded observations at minute-level intervals, while event markers were additionally assigned to critical operational stages, including vehicle departure, loading and unloading, intermediate stops, and arrival, allowing dynamic variations in refrigeration load and equipment abnormalities under extreme heat conditions to be characterized. Logistics information was jointly derived from vehicle GPS devices, vessel AIS systems, transportation management systems, and port operation records. The collected variables mainly included longitude and latitude, speed, heading, stopping location, route deviation, estimated arrival time, actual arrival time, port waiting time, and transportation delay. Raw trajectories were segmented according to transportation orders, and road or port nodes were incorporated to distinguish normal movement, abnormal stopping, congestion, and disruption states, thereby enabling climate-induced disturbances to be mapped onto the transportation process. Trade and contextual information was primarily obtained from the digital business systems of cooperating trading enterprises, logistics-order platforms, customs declaration and clearance records, and publicly available policy and market information sources. Each trade event was associated with fields including contracts, orders, invoices, customs declarations, commodity names, quantities, prices, declared values, origins, destinations, scheduled delivery times, and actual fulfillment status. Changes in trade policies, transportation restrictions, extreme-weather warnings, and market prices of major agricultural commodities were also recorded.

#### 3.1.1. Ground Truth Labeling Criteria, Business Basis, and Lookahead Horizon

To ensure objective, verifiable ground truth supervision for trade risk classification, explicit quantitative business criteria were established in collaboration with participating enterprise operators and logistics auditors. For each temporal observation window $W_{k}=\left[t_{k}-L,t_{k}\right]$ (L=6h, anchored at forecast timestamp $t_{k}$), a ground-truth supervision label $y_{k}\in \left\{0,1,2\right\}$ is defined over the forward lookahead prediction horizon $\left(t_{k},t_{k}+\Delta T\right]$ (ΔT=24h), predicting whether a commercial disruption manifests within the subsequent 24 h. Crucially, all operational outcomes occurring after $t_{k}$ (such as arrival decay inspection, customs clearance holds, or payment dispute settlements) are strictly masked from the model’s feature input at $t_{k}$, guaranteeing strict temporal causality without label leakage. Candidate windows lacking a verified 24-h follow-up disposition record (e.g., due to premature tracking termination) were completely removed (480 windows). If multiple risk symptoms occur within the 24-h horizon, the overall window label is assigned according to the maximum severity across all five operational risk dimensions:

(1) $$y_{k}=\max\left(s_{\mathrm{qual}},s_{\mathrm{delay}},s_{\mathrm{cold}},s_{\mathrm{reg}},s_{\mathrm{trade}}\right)\in \left\{0,1,2\right\},$$

where each dimensional sub-score $s_{\left(\cdot \right)}\in \left\{0,1,2\right\}$ is mapped according to mutually exclusive, complete quantitative thresholds:

Specifically, operational criteria are structured as follows: (1) *Cargo Quality Loss ($s_{\mathrm{qual}}$)*: Evaluated upon destination arrival or batch inspection: Normal ($s_{\mathrm{qual}}=0$) if unmarketable decay rate < 1.0%; Moderate Risk ($s_{\mathrm{qual}}=1$) if 1.0%≤decayrate≤3.0%; High Risk ($s_{\mathrm{qual}}=2$) if decay rate > 3.0%. (2) *Transit Delay ($s_{\mathrm{delay}}$)*: Evaluated against the contractually stipulated delivery schedule: Normal ($s_{\mathrm{delay}}=0$) if transit delay < 6.0 h; Moderate Risk ($s_{\mathrm{delay}}=1$) if 6.0h≤delay≤12.0h; High Risk ($s_{\mathrm{delay}}=2$) if delay > 12.0 h. (3) *Cold-Chain Thermal Excursions ($s_{\mathrm{cold}}$)*: Optimal temperature setpoints are calibrated to postharvest physiology (10–12 °C for Solanaceae to prevent chilling injury, 0–4 °C for Brassicaceae). To eliminate multi-condition ambiguity, temperature deviation severity $s_{\Delta T}$ and degree-hours severity $s_{\mathrm{DH}}$ are evaluated independently before taking their maximum: $s_{\mathrm{cold}}=\max\left(s_{\Delta T},s_{\mathrm{DH}}\right)$. Specifically, $s_{\Delta T}=0$ if temperature departure ΔT<2.0°C, 1 if 2.0°C≤ΔT<4.0°C, and 2 if ΔT≥4.0°C. Cumulative exposure severity $s_{\mathrm{DH}}=0$ if degree-hours DH<10.0°C·h, 1 if 10.0°C·h≤DH<40.0°C·h, and 2 if DH≥40.0°C·h. (4) *Regulatory and Customs Status ($s_{\mathrm{reg}}$)*: Normal ($s_{\mathrm{reg}}=0$) for routine green-channel clearance with zero inspection delay; Moderate Risk ($s_{\mathrm{reg}}=1$) for secondary physical inspection or documentation re-audit causing clearance delay <6.0h without detention; High Risk ($s_{\mathrm{reg}}=2$) for quarantine detention, phytosanitary hold ≥6.0h, laboratory sampling failure, border rejection, or customs confiscation. (5) *Contractual Dispute and Claim ($s_{\mathrm{trade}}$)*: Normal ($s_{\mathrm{trade}}=0$) for clean bill of lading and full invoice settlement; Moderate Risk ($s_{\mathrm{trade}}=1$) for price concession or commercial quality deduction claim <5% of invoice value; High Risk ($s_{\mathrm{trade}}=2$) for cargo rejection, order cancellation, formal insurance claim ≥5% of shipment value, or legal dispute. Under these mutually exclusive criteria, each temporal window is mapped to one of three overall tiers: *Normal/Low Risk* (Class 0, 64.5%, 24,768 windows), *Moderate Risk* (Class 1, 23.2%, 8909 windows), and *High Risk* (Class 2, 12.3%, 4723 windows).

Ground truth labeling was audited by a panel of three independent domain experts: a cold-chain logistics operations director, an international agricultural trade compliance auditor, and a postharvest quality inspector. The panel cross-referenced physical telemetry with digitized ERP records, port clearance logs, and insurance claims. Inter-annotator agreement achieved Fleiss’ Kappa κ=0.88, denoting substantial consensus, with marginal discrepancies reconciled through unanimous case adjudication.

#### 3.1.2. Data Ethics, Privacy Protection, and Compliance

The collected dataset incorporates commercial business records, trade contracts, logistics manifests, and customs declarations containing sensitive proprietary information. To ensure strict compliance with the Personal Information Protection Law (PIPL) and international data governance frameworks, a three-stage privacy-preserving pipeline was executed in sequential order prior to model training: (1) *Cryptographic Pseudonymization*: All categorical PII and corporate identifiers (enterprise legal names, tax registration codes, driver identities, vehicle license plates, and vessel MMSI numbers) were stripped and mapped to irreversible pseudo-anonymous tokens using salted SHA-256 cryptographic hashing. (2) *Financial Rescaling*: Specific monetary transaction values (invoice totals, contract valuations, claim settlements, and penalty amounts) were transformed into unitless relative percentages normalized against order-level baselines, completely eliminating absolute commercial values while preserving contractual risk gradients. (3) *Spatial Perturbation*: Geographical coordinates within private farming zones and origin packing facilities were perturbed with controlled Gaussian noise (σ=0.01°≈1.1km), masking precise plot boundaries while preserving macro-corridor transit topologies. Formal data usage authorizations and NDAs were executed with all participating entities. The open-source code and a de-identified benchmark sample (1000 representative multimodal observation windows) are publicly accessible at https://github.com/Aurelius-04/AgriClimate-EdgeNet.git (accessed on 23 September 2026). The complete enterprise dataset containing multi-year proprietary records is accessible for non-commercial academic research upon request from the corresponding author subject to an institutional NDA.

**Table 1:** Composition and scale of the multimodal agricultural trade dataset under extreme climate conditions.

Data Type	Sensor Model/Data Source	Data Volume
Climate and meteorological environmental data	Campbell Scientific CR1000X/ERA5	286,400 records
Farmland soil and production environmental data	Decagon 5TE	412,800 records
Fixed crop visual images	Hikvision DS-2CD	24,760 images
UAV agricultural imagery	DJI Mavic 3 Multispectral	8540 images
Postharvest commodity and packaging images	Sony α7 IV	13,860 images
Internal quality and Vis-NIR spectral scans	Ocean Insight Maya2000 Pro	18,200 scans
Cold-chain temperature, humidity, and warehousing status data	Sensirion SHT35	365,200 records
Vehicle GPS trajectory data	Queclink GV300	126,840 trajectory segments
Vessel AIS and port logistics data	AIS Class A Terminal	72,610 records
Trade documents and fulfillment records	Digital trade and customs declaration platform	31,480 documents
Policy, weather-warning, and market-context data	Official information platforms/API	18,760 records

Manufacturers: Campbell Scientific (Logan, UT, USA); Decagon Devices (Pullman, WA, USA); Hikvision (Hangzhou, China); DJI (Shenzhen, China); Sony (Tokyo, Japan); Ocean Insight (Orlando, FL, USA); Sensirion (Staefa, Switzerland); Queclink (Shanghai, China).

### 3.2. Data Preprocessing and Extreme-Climate Augmentation

Multimodal agricultural trade data are collected from meteorological stations, field sensors, GPS/AIS systems, cold-chain devices, visual terminals, and trade information systems, resulting in substantial heterogeneity in sampling frequency, temporal scale, and data structure. To reduce the adverse effects of temporal misalignment, scale inconsistency, and abnormal noise on model training, temporal alignment, modality-specific preprocessing, and extreme-climate-driven data augmentation are sequentially performed. These operations provide standardized and robust inputs for the subsequent cross-modal consistency modeling process.

#### 3.2.1. Temporal Alignment

The fundamental objective of temporal alignment is to map asynchronous observations with disparate sampling frequencies into a unified temporal representation while strictly respecting temporal causality. Let $t_{k}=t_{0}+k\cdot S$ denote the *k*-th forecast decision timestamp, where S=1h is the sliding step. The historical observation window preceding $t_{k}$ is formally defined as $W_{k}=\left[t_{k}-L,t_{k}\right]$ with length L=6h. The downstream risk prediction task targets commercial disruption within the forward lookahead horizon $\left(t_{k},t_{k}+\Delta T\right]$ with ΔT=24h. Crucially, all feature processing and data ingestion at decision point $t_{k}$ strictly enforce the causal constraint $t\leq t_{k}$, prohibiting any acausal leakage of future observations.

Within each observation window $W_{k}=\left[t_{k}-L,t_{k}\right]$, heterogeneous modalities are aligned onto a uniform 5-min temporal grid ${\left\{\tau_{j}\right\}}_{j=1}^{T_{w}}$ ($T_{w}=L/\Delta t=72$ steps for Δt=5min, with $\tau_{j}=t_{k}-L+j\Delta t\leq t_{k}$). High-frequency sensor streams (e.g., GPS velocity, refrigeration compressor states) falling within each 5-min interval are aggregated using causal mean pooling. For observations with irregular or lower sampling rates (e.g., hourly meteorological updates or event-driven documentation), feature alignment is performed strictly causally by retrieving the most recent observation recorded prior to or at time $\tau_{j}$:

(2) $$x_{\tau_{j}}=x_{i^{*}},\;i^{*}=\arg\max_{i:t_{i}\leq \tau_{j}}t_{i}.$$

When sensory dropouts or telemetry communication interruptions occur, missing values are imputed strictly causally via forward-fill from the last observed measurement at or before $\tau_{j}$; if no prior observation exists within the window, the entry is zero-imputed. A binary missing mask $m_{\tau_{j}}\in \left\{0,1\right\}$ and the elapsed time $\Delta \tau_{j}=\tau_{j}-t_{\mathrm{last}}$ since the last valid observation are concatenated to the feature vector, enabling downstream attention layers to explicitly perceive sensory missingness without relying on acausal future interpolation.

#### 3.2.2. Modality-Specific Preprocessing

The principle of modality-specific preprocessing is to transform heterogeneous observations into representations suitable for neural-network learning while preserving the physical and semantic characteristics of each modality. Numerical signals derived from meteorological stations, field sensors, and cold-chain devices are first subjected to outlier removal and standardization:

(3) $$x_{t}^{\prime }=\frac{x_{t}-\mu }{\sigma },$$

where μ and σ denote the mean and standard deviation calculated exclusively from the training data, respectively. Temporal differences $\Delta x_{t}=x_{t}-x_{t-1}$ and local trend features are further calculated to characterize short-term variations in temperature, humidity, soil moisture, and logistics states. Visual observations are resized to a unified spatial resolution and processed through normalization, random cropping, and flipping. Trade documents and policy-related texts are transformed into semantic representations using a unified text encoder. GPS and AIS trajectories are subjected to trajectory cleaning, abnormal-position removal, and transportation-stage segmentation, thereby facilitating the identification of abnormal stops, route deviations, and transportation delays.

#### 3.2.3. Extreme-Climate-Driven Data Augmentation

Extreme-climate samples are generally substantially less frequent than normal-condition samples. Therefore, a data augmentation strategy constrained by agricultural trade risk propagation is designed instead of applying independent random perturbations only to meteorological variables. Crucially, different categories of extreme meteorological hazards exert heterogeneous, stage-specific physical and operational impacts across the agricultural supply chain: (1) *Extreme Heatwaves*: In the production stage, extreme heat triggers severe canopy transpiration stress and soil moisture depletion; in the commodity stage, it accelerates postharvest enzymatic respiration, ethylene synthesis, and pulp softening; in the cold-chain stage, high ambient temperatures degrade condenser heat-exchange efficiency, elevating compressor refrigeration duty cycles by 35–50% and increasing door-opening temperature recovery times by over 40%; in the logistics stage, tire blowout hazards and engine overheating necessitate reduced daytime travel speeds; and in the trade stage, supply shrinkage induces rapid wholesale price surges. (2) *Heavy Rainfall and Flash Flooding*: In the production stage, torrential downpours cause field waterlogging, root hypoxia, and disease outbreaks; in the commodity stage, excess humidity causes packaging cardboard softening, moisture condensation, and fungal rot; in the cold-chain stage, door seal condensation and relative humidity spikes disrupt chamber microclimates; in the logistics stage, arterial highway submergence and bridge washouts reduce average fleet speeds by 45–65%, multiply detour distances by 30–80%, and induce severe transshipment port congestion; and in the trade stage, delivery deadlines are breached, prompting force majeure claims and contract renegotiations. (3) *Coastal Gales and Typhoons*: Structural lodging in production fields combines with suspended crane operations at container terminals and mandatory vessel AIS mooring delays (24–72 h), directly halting intermodal logistics.

The augmented representation of modality *m* under climate hazard *c* is formulated as

(4) $${\tilde{x}}^{\left(m\right)}=x^{\left(m\right)}+\lambda_{m}d_{c}^{\left(m\right)}+\epsilon^{\left(m\right)},$$

where the climate-conditioned perturbation vector $d_{c}^{\left(m\right)}$ governs the cross-stage response direction, and $\lambda_{m}$ modulates the hazard impact intensity. The parameter values of $d_{c}^{\left(m\right)}$ were empirically estimated from historical multi-hazard disruption records during the training partition (May 2023 to October 2024, comprising *N* = 4210 anomalous transport windows) alongside historical regional disaster archives from the China Meteorological Data Service Centre (CMDC, https://data.cma.cn/ (accessed on 23 September 2026)) and the international Emergency Events Database (EM-DAT, https://www.emdat.be/ (accessed on 23 September 2026)) across 12 severe events in North China (including the torrential flooding of July 2023 and the severe heatwaves of June–July 2023). Specifically, for flood hazards, the empirical logistics perturbation vector $d_{\mathrm{flood}}^{\left(\mathrm{logistics}\right)}$ encodes a 52.0% fleet velocity reduction (median highway speed dropping from 68.4km/h to 32.8km/h, p<0.001) coupled with a 3.5-fold increase in route stagnation dwell time (0.6h to 2.1h); for heatwaves, the cold-chain vector $d_{\mathrm{heat}}^{\left(\mathrm{cold}\_\mathrm{chain}\right)}$ encodes a +3.8°C compartment thermal excursion with a 45.2% prolonged temperature pulldown duration. The scaling coefficient $\lambda_{m}$ is determined piecewise linearly from historical meteorological anomaly *z*-scores ($z=\left(x_{\mathrm{met}}-{\bar{x}}_{\mathrm{met}}\right)/s_{\mathrm{met}}$): $\lambda_{m}=0$ for $z<z_{90}=1.282$ (routine variation); $\lambda_{m}=1.0$ at the 95th percentile ($z_{95}=1.645$); and $\lambda_{m}=1.5$ at the 99th percentile ($z_{99}=2.326$). The stochastic term $\epsilon^{\left(m\right)}∼N\left(0,\sigma_{m}^{2}\right)$, with $\sigma_{m}\leq 0.05\mathrm{std}\left(X^{\left(m\right)}\right)$, injects localized sensor jitter to ensure manifold smoothness.

*Prediction-Horizon Constraint on Augmentation*: The target remains the maximum risk severity in the future interval $\left(t_{k},t_{k}+24\;h\right]$, whereas model inputs are restricted to the historical interval $\left[t_{k}-6\;h,t_{k}\right]$. Measurement-level corruption, such as missing observations or sensor noise, does not itself alter the underlying commercial outcome, so the observed future label is retained for outcome-preserving perturbations. A physical hazard intervention is different: perturbing the historical input alone does not determine a new future label. Valid relabeling would require a paired synthetic future trajectory and corresponding commercial outcomes over the same 24-h horizon, with all five severity dimensions evaluated there:

(5) $${\tilde{y}}_{k}=\max\left({\tilde{s}}_{\mathrm{qual}}^{\left(t_{k},t_{k}+24h\right]},{\tilde{s}}_{\mathrm{delay}}^{\left(t_{k},t_{k}+24h\right]},{\tilde{s}}_{\mathrm{cold}}^{\left(t_{k},t_{k}+24h\right]},{\tilde{s}}_{\mathrm{reg}}^{\left(t_{k},t_{k}+24h\right]},{\tilde{s}}_{\mathrm{trade}}^{\left(t_{k},t_{k}+24h\right]}\right).$$

For example, the High-Risk thermal criterion is degree-hours of at least 40.0°C·h within the future outcome interval, not a threshold crossed only in the perturbed historical input. The present description does not establish a validated generator of paired future physical and commercial outcomes. Consequently, severe hazard perturbations cannot, on their own, be treated as verified new ground-truth events, and the augmentation results do not establish the validity of synthetic future-risk relabeling. Furthermore, random modality masking, temporal masking, sensor noise injection, trajectory perturbation, and modality dropout are employed to simulate sensor failures, communication interruptions, incomplete observations, and trajectory disturbances. Through these operations, the robustness of AgriClimate-EdgeNet under extreme climate conditions and incomplete multimodal sensing environments is enhanced.

### 3.3. Proposed Method

#### 3.3.1. Overall

After unified temporal alignment and standardization, the processed climate and environmental data, agricultural production information, commodity visual observations, cold-chain states, logistics trajectories, and trade information are first fed into their corresponding modality-specific encoders to extract temporal and semantic features within a unified representation space. The resulting multimodal representations are subsequently passed to the extreme-climate-aware cross-modal consistency module, in which climate conditions are treated as contextual constraints to jointly model key relationships, including climate–production, climate–logistics, production–trade, visual–document, cold chain–commodity, and logistics–document. Through cross-modal interaction and consistency matching, consistency scores, conflict representations, and climate-impact representations are obtained, thereby characterizing the potential propagation of extreme climate shocks throughout the agricultural trade chain. These representations are then delivered to the lightweight edge temporal risk prediction module, where depthwise separable temporal convolution, gated temporal units, and lightweight attention are employed to capture short-term dynamic variations, while the consistency scores are used to regulate the contribution of different information sources during feature fusion. A cloud-based Teacher model further constrains the edge-based Student model through knowledge distillation, allowing informative risk representations to be retained under reduced computational overhead. Subsequently, the reliability- and uncertainty-aware robust risk inference module estimates dynamic reliability weights according to modality missingness, noise, sampling delay, and signal stability, recalibrates the fused representation, and quantifies the confidence of the final prediction. Trade risk probability, risk level, predictive uncertainty, and the dominant risk source are ultimately generated, forming a complete closed-loop framework from extreme-climate impact perception and cross-modal conflict identification to real-time edge risk warning.

#### 3.3.2. Extreme-Climate-Aware Cross-Modal Consistency Module

Figure 1 illustrates the extreme-climate-aware cross-modal consistency module. The heterogeneous observations across the six trade chain modalities are first mapped into a unified latent space of width d=128 through dedicated modality encoders: (1) *Climate and Environmental Sequence* ($X_{c}\in R^{T\times C_{c}}$, $T=72,C_{c}=8$ meteorological features): Encoded via a 2-layer 1D CNN with gated residual units. (2) *Agricultural Production Sequence* ($X_{p}\in R^{T\times C_{p}}$, $T=72,C_{p}=6$ Soil and canopy features): Encoded via a 2-layer 1D CNN with gated residual units. (3) *Commodity Status Modality* ($X_{\mathrm{comm}}$): Incorporates both RGB surface imagery $X_{\mathrm{rgb}}\in R^{224\times 224\times 3}$ and Vis-NIR diffuse reflectance spectra $x_{\mathrm{spec}}\in R^{512}$ (350–1050 nm). Following Standard Normal Variate (SNV) transformation and first-order Savitzky-Golay filtering, the spectral scan is processed by an on-device 3-layer 1D CNN (kernel sizes 5, 3, 3; channels 32, 64, 128) with global average pooling to yield a 128-d spectral latent embedding $h_{\mathrm{spec}}$. Simultaneously, RGB images are encoded by a lightweight MobileNetV3 backbone to yield visual embedding $h_{\mathrm{rgb}}\in R^{128}$. A sigmoid-gated feature fusion layer combines internal spectral and superficial visual features: $g_{\mathrm{comm}}=\sigma \left(W_{g}\left[h_{\mathrm{rgb}};h_{\mathrm{spec}}\right]+b_{g}\right)$, yielding commodity representation $h_{\mathrm{comm}}=g_{\mathrm{comm}}⊙h_{\mathrm{rgb}}+\left(1-g_{\mathrm{comm}}\right)⊙h_{\mathrm{spec}}$. All baseline models and the Commodity Status ablation variant receive identical visual-spectral representations to ensure benchmark fairness. (4) *Cold-Chain Sequence* ($X_{cc}\in R^{T\times C_{cc}}$, $T=72,C_{cc}=4$ reefer temperature, humidity, and duty cycles): Encoded via an on-device 2-layer 1D CNN with gated residual units. (5) *Logistics Trajectory* ($X_{\log}\in R^{T\times C_{\log}}$, $T=72,C_{\log}=5$ GPS speed, heading, dwell, and distance features): Encoded via an on-device 2-layer 1D CNN with gated residual units. (6) *Trade and Regulatory Context*: Processed by a 2-layer DistilBERT encoder fine-tuned on customs declarations and trade contracts to generate text embeddings. In operational edge deployment aboard the NVIDIA Jetson AGX Orin, textual trade contracts and packing-line RGB inspection images undergo asynchronous, offline feature extraction at origin intake (caching static 128-d embeddings), whereas real-time streaming sensory sequences (Climate, Production IoT, Reefer Telemetry, Vehicle GPS) and Vis-NIR portable spectrometer scans are processed directly on-device. The reported 3.96M parameters, 1.21G FLOPs, and 8.6ms latency comprehensively encompass all on-device encoders, consistency cross-attention, lightweight temporal blocks, and reliability-uncertainty inference networks. Each modality representation is projected into a *d*-dimensional latent sequence:

(6) $$Z_{m}=\phi_{m}\left(X_{m};\Theta_{m}\right)W_{m},\;Z_{m}\in R^{T^{\prime }\times d}.$$

To enable cross-modal relationships to adapt dynamically to extreme climate conditions, a climate-conditioned vector $g_{c}$ is first generated from the climate representation and injected into the climate–production, climate–logistics, production–trade, visual–document, cold chain–commodity, and logistics–document relationships.

To prevent the position-invariant climate condition vector from being mathematically cancelled out by the Softmax normalization across key positions (which occurs when an identical vector is added uniformly across all keys), climate conditioning is formulated as Feature-wise Linear Modulation (FiLM) channel gating over Keys and Values:

(7) $${\tilde{K}}_{q}=\left(Z_{q}W_{q}^{K}\right)⊙\left(1+\tanh(g_{c}W_{c,\gamma }^{K})\right),\;{\tilde{V}}_{q}=\left(Z_{q}W_{q}^{V}\right)⊙\left(1+\tanh(g_{c}W_{c,\gamma }^{V})\right),$$

where ⊙ denotes the element-wise Hadamard product, and $W_{c,\gamma }^{K},W_{c,\gamma }^{V}\in R^{d\times d}$ project the climate condition vector $g_{c}$ into channel-wise scaling modulators. This channel-wise modulation dynamically scales feature dimensions according to the climate hazard profile prior to the dot product, making the query-key affinity sensitive to environmental stress. The climate-conditioned cross-modal attention is then computed as

(8) $$A_{pq}=\mathrm{softmax}\left(\frac{\left(Z_{p}W_{p}^{Q}\right){\tilde{K}}_{q}^{⊤}}{\sqrt{d}}\right),\;R_{pq}=A_{pq}{\tilde{V}}_{q}.$$

Setting $g_{c}=0$ yields tanh(0)=0, providing a mathematically neutral scaling factor of 1 that naturally recovers standard unconditioned cross-attention without architectural modification. A normalized relation distance is subsequently employed to quantify cross-modal consistency, such that correctly matched samples are encouraged to approach each other in the latent space, whereas abnormal combinations generated through modality shuffling are pushed apart:

(9) $$s_{pq}=\exp\left[-\frac{{∥{\hat{Z}}_{p}-{\hat{R}}_{pq}∥}_{2}^{2}}{\tau_{s}}\right],\;L_{con}=-\log\frac{\exp(s_{pq}^{+}/\tau_{c})}{\exp\left(s_{pq}^{+}/\tau_{c}\right)+\sum_{j}\exp\left(s_{pq,j}^{-}/\tau_{c}\right)},$$

where $\tau_{s}>0$ denotes the spatial Gaussian kernel bandwidth (empirically set to $\tau_{s}=2.0$) governing the metric distance sensitivity in the latent space, whereas $\tau_{c}>0$ represents the distinct contrastive temperature hyperparameter in the InfoNCE loss (set to $\tau_{c}=0.07$) regulating the sharpness of cross-modal representation discrimination. Cross-modal inconsistency is further explicitly represented as

(10) $$F_{pq}=\left(1-s_{pq}\right)\left|{\hat{Z}}_{p}-{\hat{R}}_{pq}\right|,\;E_{clim}=\sum_{(p,q)\in P}\alpha_{pq}\left[R_{pq}∥F_{pq}∥g_{c}\right],$$

where P denotes the predefined set of cross-modal relationships and $\alpha_{pq}$ is adaptively learned through relation-level attention. Since the squared Euclidean distance always satisfies ${∥{\hat{Z}}_{p}-{\hat{R}}_{pq}∥}_{2}^{2}\geq 0$, the consistency score necessarily satisfies $0<s_{pq}\leq 1$. Furthermore, differentiation with respect to the relation distance $D_{pq}$ gives

(11) $$\frac{\partial s_{pq}}{\partial D_{pq}}=-\frac{1}{\tau_{s}}\exp\left(-\frac{D_{pq}}{\tau_{s}}\right)<0.$$

This property demonstrates that the consistency score decreases strictly monotonically as the cross-modal discrepancy increases, allowing production abnormalities, cold-chain variations, and logistics delays induced by extreme climate events to be converted into continuously learnable conflict intensities. The resulting consistency scores, conflict representations, and climate-impact representations are jointly passed to the subsequent lightweight edge temporal module. Consequently, information from individual modalities is not only exploited independently, but the propagation relationship of “climate shock–state variation–information conflict” across the agricultural trade chain is also explicitly perceived, thereby improving risk identification under extreme climate conditions and abnormal trade states.

#### 3.3.3. Reliability- and Uncertainty-Aware Robust Risk Inference Module

The reliability- and uncertainty-aware robust risk inference module receives the multimodal risk representations generated by the lightweight edge temporal module and performs quality-aware recalibration of heterogeneous information sources before final prediction.

As shown in Figure 2, let the input representation of the *m*-th modality be $F_{m}\in R^{H_{m}\times W_{m}\times C_{m}}$. Feature compression is first performed by a reliability estimation network composed of $L_{r}$ layers. The first $L_{r}-1$ layers consist of linear transformations or 1×1 convolutions with channel width $C_{r}^{\left(l\right)}$, followed by normalization and nonlinear activation, whereas the final layer maps the representation into a scalar reliability space. The quality descriptor $u_{m}={\left[q_{m},n_{m},d_{m},s_{m},e_{m}\right]}^{⊤}$ integrates five standardized quality metrics strictly bounded in [0,1]: (1) missing rate $q_{m}=\frac{1}{T_{m}C_{m}}\sum_{t=1}^{T_{m}}\sum_{c=1}^{C_{m}}M_{t,c}^{\left(m\right)}\in \left[0,1\right]$, where $M_{t,c}^{\left(m\right)}\in \left\{0,1\right\}$ denotes the raw missingness bitmask before zero or forward-fill imputation; (2) noise intensity $n_{m}=\frac{1}{C_{m}}\sum_{c=1}^{C_{m}}\tanh\left(\frac{{\hat{\sigma }}_{c}}{\sigma_{\mathrm{nominal},c}}\right)\in \left[0,1\right)$, where the second-order difference filters out slow physical diurnal trends to isolate sensor electrical jitter and measurement turbulence ${\hat{\sigma }}_{c}$, normalized against nominal baseline variance $\sigma_{\mathrm{nominal},c}$; (3) sampling delay $d_{m}=\min\left(1,\frac{t_{\mathrm{current}}-t_{\mathrm{latest}}^{\left(m\right)}}{T_{\max}}\right)\in \left[0,1\right]$, normalized by the maximum tolerable buffer latency threshold $T_{\max}=60\;\min$; (4) signal stability $s_{m}=\exp\left(-\gamma \frac{\mathrm{std}(X^{\left(m\right)})}{\mathrm{mean}(|X^{\left(m\right)}|)+\epsilon }\right)\in \left[0,1\right]$, capturing the inverse of the temporal coefficient of variation with scaling parameter γ=0.5; (5) historical prediction error $e_{m}=\frac{1}{W}\sum_{w=1}^{W}\frac{1}{2}{∥p_{w}^{\left(m\right)}-y_{w}∥}_{2}^{2}\in \left[0,1\right]$, evaluated via the normalized Brier score of unimodal predictions across W=20 historically closed and audited shipment orders. For non-time-series modalities, equivalent bounded quality descriptors are computed: for visual images, $q_{\mathrm{vis}}$ represents the camera frame dropout ratio, $n_{\mathrm{vis}}=\tanh\left(\sigma_{\mathrm{Laplace}}/\sigma_{0}\right)$ captures high-frequency blur and sensor noise, $d_{\mathrm{vis}}$ denotes image transmission latency, $s_{\mathrm{vis}}$ measures inter-frame illumination consistency, and $e_{\mathrm{vis}}$ is the Brier score of the visual classifier; for trade text documents, $q_{\mathrm{text}}$ quantifies missing mandatory declaration fields, $n_{\mathrm{text}}=\tanh\left(\mathrm{OCR}\;\mathrm{character}\;\mathrm{entropy}\;\mathrm{anomaly}\right)$, $d_{\mathrm{text}}$ measures document filing delay, $s_{\mathrm{text}}$ denotes structural schema conformity, and $e_{\mathrm{text}}$ is the Brier score of the text classifier.

**Figure 1:**
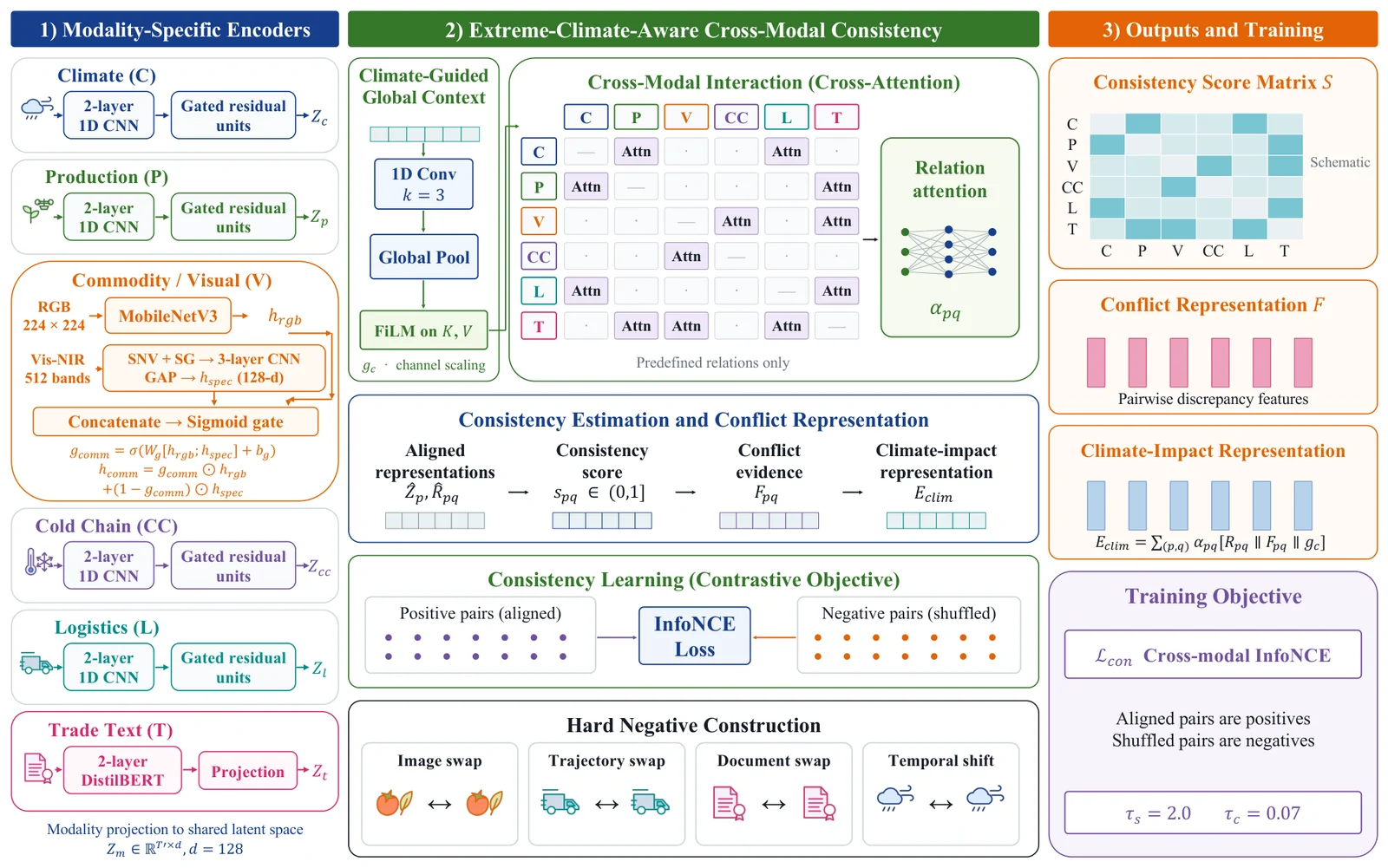
The extreme-climate-aware cross-modal consistency module integrates modality-specific encoding, climate-guided cross-modal interaction, and contrastive consistency learning to derive consistency scores, conflict representations, and climate-impact representations.

The modality reliability score is formulated as

(12) $$r_{m}=\sigma \left(w_{r}^{⊤}\rho \left(W_{r}\left[\mathrm{Pool}\left(F_{m}\right)∥u_{m}\right]+b_{r}^{\left(1\right)}\right)+b_{r}^{\left(2\right)}\right),$$

where ρ(·) denotes a nonlinear activation, σ(·) is the sigmoid function constraining $r_{m}\in \left(0,1\right)$, $b_{r}^{\left(1\right)}\in R^{C_{r}^{\left(1\right)}}$ represents the intermediate linear layer bias vector, and $b_{r}^{\left(2\right)}\in R$ represents the scalar bias of the final output projection. To simultaneously exploit the cross-modal consistency information supplied by the preceding module, the aggregate consistency response of modality *m* is formulated as $c_{m}=\frac{1}{|P_{m}|}\sum_{q\in P_{m}}s_{mq}\in \left(0,1\right]$, where $P_{m}$ denotes the subset of cross-modal pairs involving modality *m*. Normalized gating is then applied to determine the dynamic contribution of each modality:

(13) $$\omega_{m}=\frac{{\left(r_{m}+\epsilon \right)}^{\alpha }{\left(c_{m}+\epsilon \right)}^{\beta }}{\sum_{j\in M}{\left(r_{j}+\epsilon \right)}^{\alpha }{\left(c_{j}+\epsilon \right)}^{\beta }},\;\tilde{F}=\sum_{m\in M}\omega_{m}P_{m}\left(F_{m}\right),$$

where $P_{m}$ projects each modality into a unified risk representation space with width $d_{f}$. The exponential hyperparameters α and β govern the sensitivity of dynamic weighting to intrinsic sensing quality $r_{m}$ and semantic cross-modal consistency $c_{m}$, respectively. Through grid-search calibration on the validation split over α,β∈{0.5,1.0,1.5,2.0}, setting α=1.0 and β=1.0 achieved the optimal trade-off between Macro-F1 and ECE, ensuring that sensor-level physical corruption and cross-stage relational conflicts are penalized symmetrically without premature modality collapse.

Since $\omega_{m}>0$ and $\sum_{m}\omega_{m}=1$, the fused representation always lies within the convex hull formed by the modality-specific projected representations. To rigorously establish the sensitivity of $\omega_{m}$ with respect to $r_{m}$ under the ceteris paribus condition that other modality reliability scores $r_{j\neq m}$ and consistency scores $c_{k}$ remain fixed, let $v_{j}={\left(r_{j}+\epsilon \right)}^{\alpha }{\left(c_{j}+\epsilon \right)}^{\beta }$ and define the denominator sum $S=\sum_{k\in M}v_{k}$. With other variables held constant, $\frac{\partial v_{m}}{\partial r_{m}}=\frac{\alpha v_{m}}{r_{m}+\epsilon }$ and $\frac{\partial v_{k}}{\partial r_{m}}=0$ for all k≠m, yielding $\frac{\partial S}{\partial r_{m}}=\frac{\partial v_{m}}{\partial r_{m}}=\frac{\alpha v_{m}}{r_{m}+\epsilon }$. Applying the quotient rule to $\omega_{m}=\frac{v_{m}}{S}$, we get

(14) $$\frac{\partial \omega_{m}}{\partial r_{m}}=\frac{\frac{\partial v_{m}}{\partial r_{m}}S-v_{m}\frac{\partial S}{\partial r_{m}}}{S^{2}}=\frac{\frac{\alpha v_{m}}{r_{m}+\epsilon }S-v_{m}\left(\frac{\alpha v_{m}}{r_{m}+\epsilon }\right)}{S^{2}}=\frac{\alpha \omega_{m}\left(1-\omega_{m}\right)}{r_{m}+\epsilon }>0.$$

This result demonstrates that the contribution of a modality increases strictly monotonically with its reliability. Conversely, when missing observations, noise, or sampling delays reduce $r_{m}$, the corresponding information is automatically suppressed, thereby limiting the influence of unreliable sensing signals on the final decision.

**Figure 2:**
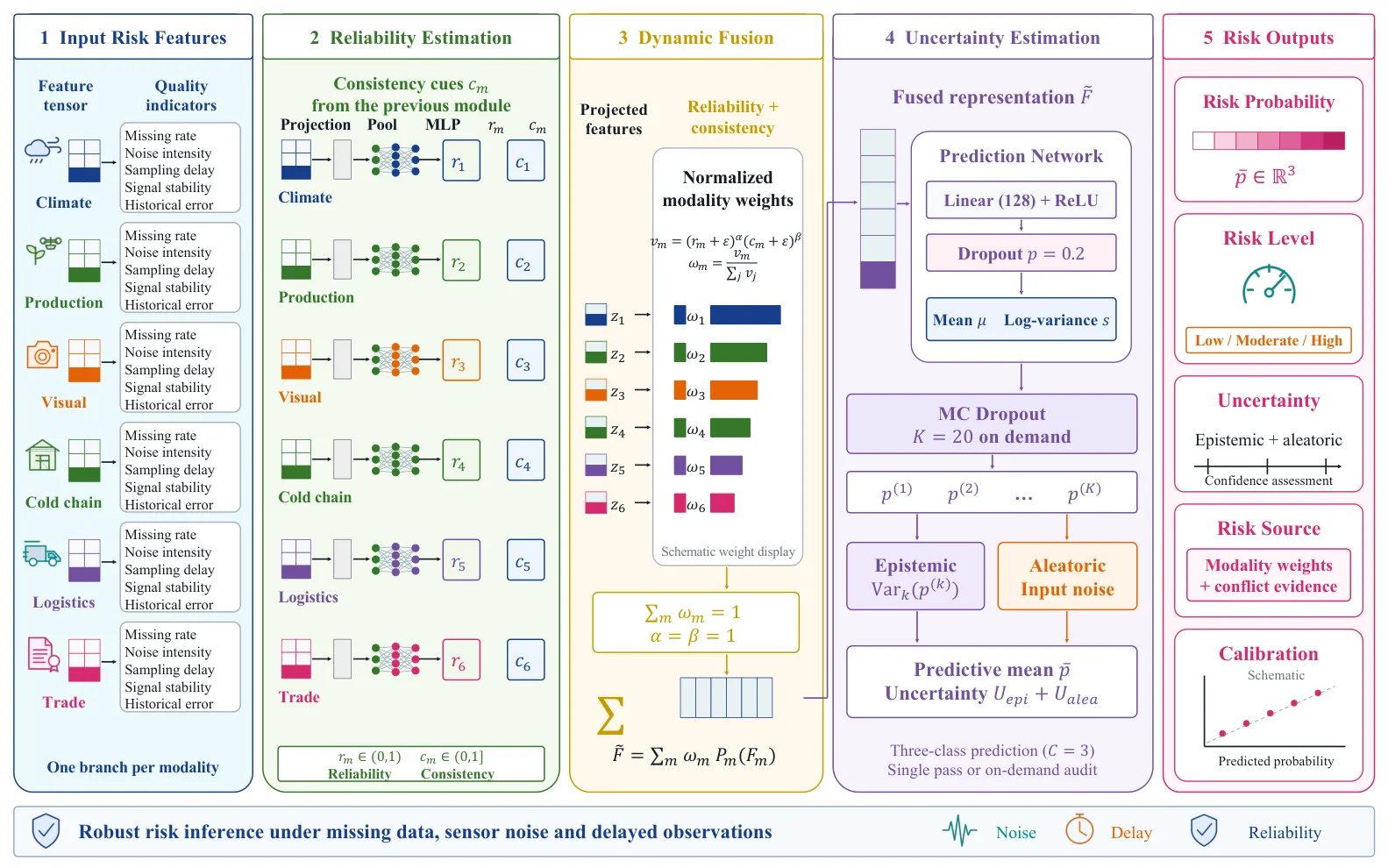
The reliability- and uncertainty-aware robust risk inference module integrates modality reliability estimation, dynamic weighted fusion, and stochastic uncertainty inference to jointly output risk probability, risk level, predictive uncertainty, and major risk sources.

To prevent overconfident predictions during sensory degradation and adverse meteorological conditions, a dual uncertainty estimation mechanism is constructed that simultaneously models epistemic model uncertainty and heteroscedastic aleatoric uncertainty. The uncertainty prediction head predicts class mean logits $\mu \in R^{C}$ and heteroscedastic observation log-variance $s=\log\sigma^{2}\in R^{C}$ from $\tilde{F}$. During training, the network is optimized with an explicit heteroscedastic classification loss using Monte Carlo sampling:

(15) $$L_{\mathrm{alea}}=\frac{1}{B}\sum_{i=1}^{B}\left[-\log\left(\frac{1}{S}\sum_{s=1}^{S}\mathrm{softmax}{\left(\mu_{i}+\sigma_{i}⊙\epsilon^{\left(s\right)}\right)}_{y_{i}}\right)\right],\;\epsilon^{\left(s\right)}∼N\left(0,I\right),$$

where S=10 stochastic samples are drawn during backpropagation. When an input is corrupted or noisy, the model dynamically increases the predicted log-variance $s_{i}=\log\sigma_{i}^{2}$, softening the softmax probabilities to avoid steep cross-entropy loss penalties. To prevent unbounded variance inflation on severely corrupted or uninformative samples, the predicted log-variance is constrained via a smooth clamping activation ($s_{c}\in \left[-6.0,2.0\right]$, corresponding to variance bounds $\sigma_{c}^{2}\in \left[0.0025,7.39\right]$) and regularized with weight decay, ensuring robust numerical optimization.

During inference, epistemic and aleatoric uncertainties are decoupled to capture distinct sources of predictive variability. Epistemic uncertainty $U_{\mathrm{epi}}$ arises from model parameter ambiguity under rare or out-of-distribution extreme climate regimes, and is estimated by activating Monte Carlo Dropout ($p_{\mathrm{drop}}=0.2$) across K=20 stochastic forward passes. For each pass k∈{1,…,K}, the classification head outputs predicted logit mean $\mu^{\left(k\right)}\in R^{C}$ and heteroscedastic standard deviation $\sigma^{\left(k\right)}=\exp\left(\frac{1}{2}s^{\left(k\right)}\right)\in R^{C}$ (with variance ${\left(\sigma^{\left(k\right)}\right)}^{2}$). The predicted probability vector for pass *k* is evaluated as $p^{\left(k\right)}=\mathrm{softmax}\left(\mu^{\left(k\right)}\right)$. Note that evaluating $\mathrm{softmax}(\mu^{\left(k\right)})$ serves as a first-order Taylor approximation to the expected probability $E_{\epsilon }\left[\mathrm{softmax}\left(\mu^{\left(k\right)}+\sigma^{\left(k\right)}⊙\epsilon \right)\right]$, which holds reliably under moderate logit variance $∥\sigma^{\left(k\right)}{∥}_{2}^{2}\ll \pi^{2}/3$ and provides high computational efficiency on edge platforms. The ensemble predictive mean is

(16) $$\bar{p}=\frac{1}{K}\sum_{k=1}^{K}p^{\left(k\right)},\;U_{\mathrm{epi}}=\frac{1}{K}\sum_{k=1}^{K}{∥p^{\left(k\right)}-\bar{p}∥}_{2}^{2}.$$

Concurrently, heteroscedastic aleatoric uncertainty $U_{\mathrm{alea}}$ characterizes stochastic observation noise, sensor precision drift, and unpredictable environmental turbulence. Under the assumption of independent logit noise with variance $\mathrm{diag}({\left(\sigma^{\left(k\right)}\right)}^{2})$, the first-order multivariate Delta method propagates logit-space variance to the probability simplex through the full Softmax Jacobian matrix $J^{\left(k\right)}=\frac{\partial p^{\left(k\right)}}{\partial \mu^{\left(k\right)}}=\mathrm{diag}\left(p^{\left(k\right)}\right)-p^{\left(k\right)}{\left(p^{\left(k\right)}\right)}^{⊤}\in R^{C\times C}$. The first-order covariance approximation of the predicted probability vector is given by $\mathrm{Cov}\left(p^{\left(k\right)}\right)\approx J^{\left(k\right)}\mathrm{diag}\left({\left(\sigma^{\left(k\right)}\right)}^{2}\right){\left(J^{\left(k\right)}\right)}^{⊤}$. The scalar aleatoric uncertainty is defined as the trace of this covariance matrix (total prediction variance), averaged across the *K* Monte Carlo passes:

(17) $$U_{\mathrm{alea}}=\frac{1}{K}\sum_{k=1}^{K}\mathrm{Tr}\left(J^{\left(k\right)}\mathrm{diag}\left({\left(\sigma^{\left(k\right)}\right)}^{2}\right){\left(J^{\left(k\right)}\right)}^{⊤}\right)=\frac{1}{K}\sum_{k=1}^{K}\sum_{c=1}^{C}\sum_{j=1}^{C}{\left(J_{c,j}^{\left(k\right)}\right)}^{2}{\left(\sigma_{j}^{\left(k\right)}\right)}^{2},$$

where $J_{c,j}^{\left(k\right)}=p_{c}^{\left(k\right)}\left(\delta_{cj}-p_{j}^{\left(k\right)}\right)$. This formulation accounts for both diagonal and cross-category covariance terms. The total predictive uncertainty is computed as $U_{\mathrm{total}}=U_{\mathrm{epi}}+U_{\mathrm{alea}}$.

In operational edge deployment on the NVIDIA Jetson AGX Orin platform, AgriClimate-EdgeNet supports two distinct operational modes: (1) *Streaming Mode (Single-Pass Deterministic)*: For routine continuous monitoring aboard refrigerated vehicles and gateway terminals, the network executes a single forward pass (K=1) with Dropout deactivated, using the deterministic mean logit μ and single-pass analytical aleatoric variance. In this mode, the edge model executes in 8.6±0.3ms (116.3 inferences/s throughput), attaining an ECE of 0.028±0.003, comfortably accommodating high-rate 10–100Hz telemetry ingestion. (2) *Deep Audit Mode (K=20 MC Stochastic Passes)*: When anomalous risk signals or elevated single-pass variance is detected, the system triggers a deep uncertainty audit. Crucially, the K=20 stochastic passes are executed as a single parallelized batch tensor call (B=20) on Tensor Cores rather than sequential single inferences. The measured batched latency on Jetson AGX Orin is 38.4±1.2ms (26.0 audits/s throughput), producing a calibrated ECE of 0.021±0.002, well within the 1.0s real-time decision budget. Predictive entropy is additionally computed to characterize overall decision dispersion:

(18) $$U_{\mathrm{pred}}=-\sum_{c\in C}{\bar{p}}_{c}\log\left({\bar{p}}_{c}+\epsilon \right).$$

Because the squared norm is nonnegative, $U_{\mathrm{epi}}\geq 0$, and it becomes zero only when all stochastic inference outputs are identical. A more dispersed predictive distribution produces larger variance and entropy, allowing the uncertainty measures to reflect insufficient model knowledge under extreme climate conditions, missing modalities, or out-of-distribution samples. Finally, the risk prediction head produces the risk probability and risk level from $\tilde{F}$, while $\omega_{m}$, the cross-modal conflict response, and $U_{\mathrm{pred}}$ are jointly used to determine the dominant risk source and predictive confidence. This mechanism enables AgriClimate-EdgeNet to automatically suppress unreliable information when heavy rainfall or extreme heat induces sensor drift, communication delays, or partial modality failure, while increasing predictive uncertainty when available evidence is insufficient rather than generating erroneous high-confidence predictions. The resulting inference mechanism is therefore particularly suitable for real-time, secure, and trustworthy risk warning at distributed agricultural trade edge nodes.

#### 3.3.4. Lightweight Edge Temporal Risk Prediction Module

The lightweight edge temporal risk prediction module receives the modality-specific encoded representations, consistency scores, conflict representations, and climate-impact representations obtained from the preceding stage and organizes them into a temporal feature sequence $X\in R^{T\times C_{0}}$.

As shown in Figure 3, the network is composed of $L_{e}$ lightweight temporal blocks connected sequentially. The input of the *l*-th block has dimensions $T_{l}\times C_{l}$. A depthwise temporal convolution with kernel width $K_{l}$ and dilation rate $\delta_{l}$ is first employed to independently capture local temporal dynamics along each channel, after which pointwise convolution is applied to achieve cross-channel interaction. The corresponding outputs are defined as

(19) $$D_{t,c}^{\left(l\right)}=\sum_{k=0}^{K_{l}-1}w_{k,c}^{\left(l\right)}X_{t-\delta_{l}k,c}^{\left(l\right)},\;P_{t,j}^{\left(l\right)}=\sum_{c=1}^{C_{l}}q_{c,j}^{\left(l\right)}D_{t,c}^{\left(l\right)}.$$

A dual-branch gated structure is subsequently introduced to regulate the preservation of temporal signals associated with climate shocks, cold-chain fluctuations, and logistics anomalies:

(20) $$G^{\left(l\right)}=\tanh\left(P^{\left(l\right)}W_{g}^{\left(l\right)}\right)⊙\sigma \left(P^{\left(l\right)}W_{z}^{\left(l\right)}\right),\;X^{\left(l+1\right)}=N\left(G^{\left(l\right)}+X^{\left(l\right)}W_{s}^{\left(l\right)}\right),$$

where N(·) denotes the normalization operation, and residual projection is employed to preserve information continuity across layers with different channel widths. To avoid the high computational overhead of standard self-attention over long temporal windows, a kernel mapping φ(·) is introduced to construct lightweight temporal attention:

(21) $$O_{t}=\frac{\varphi {\left(q_{t}\right)}^{⊤}\left[\sum_{j=1}^{T}\varphi \left(k_{j}\right)v_{j}^{⊤}\right]}{\varphi {\left(q_{t}\right)}^{⊤}\left[\sum_{j=1}^{T}\varphi \left(k_{j}\right)\right]+\varepsilon }.$$

Here, following the linear attention formulation, the non-negative feature kernel map is defined as φ(x)=elu(x)+1≥0, and $\varepsilon =1\times 10^{-6}$ ensures numerical stability. Across the $L_{S}=4$ dilated temporal blocks, temporal kernel sizes are fixed at $K_{l}=3$ with dilation rates configured as $\delta_{l}\in \left\{1,2,4,8\right\}$ for l∈{1,2,3,4}, yielding an effective temporal receptive field of $R_{4}=1+\sum_{l=1}^{4}\left(3-1\right)\delta_{l}=31$ steps (155min). Combined with global linear attention across all T=72 steps (6h), this architecture jointly captures instantaneous cold-chain spikes and multi-hour cumulative trend shifts. Because the key-value statistics can be precomputed, the temporal complexity is reduced from $O(T^{2}d)$ for conventional self-attention to

(22) $$C_{\mathrm{light}}=O\left(Td^{2}\right),\;\lim_{T/d\to \infty }\frac{C_{\mathrm{light}}}{C_{\mathrm{attn}}}=\lim_{T/d\to \infty }\frac{Td^{2}}{T^{2}d}=0.$$

This relationship indicates that the computational advantage becomes increasingly pronounced as the temporal window length grows in long-sequence edge inference scenarios. Meanwhile, the effective receptive field generated by the $L_{e}$ dilated convolutional layers satisfies

(23) $$R_{L_{e}}=1+\sum_{l=1}^{L_{e}}\left(K_{l}-1\right)\delta_{l}.$$

Accordingly, instantaneous cold-chain temperature excursions, abnormal vehicle stops, and delayed propagation of climate shocks can be captured simultaneously without substantially increasing network depth. The consistency information produced by the preceding module is further converted into a temporal modulation coefficient $\eta_{t}$, which performs soft gating on the risk representation:

(24) $${\tilde{O}}_{t}=\left[\eta_{t}+\left(1-\eta_{t}\right)\lambda_{t}\right]O_{t},$$

where $\lambda_{t}$ is learned from the current conflict representation. Consequently, when cross-modal consistency decreases, abnormal information is prevented from being directly accumulated as normal evidence. During training, a cloud-based teacher network with $L_{T}$ layers and high-dimensional channel width $d_{T}$, together with an edge-based student network with $L_{S}$ layers and compact channel width $d_{S}$, is further constructed. Soft targets are employed to preserve the risk-distribution structure learned by the teacher model:

(25) $$L_{KD}=\zeta L_{\mathrm{CE}}+\left(1-\zeta \right)\Theta^{2}D_{\mathrm{KL}}\left(\pi_{T}^{\left(\Theta \right)}∥\pi_{S}^{\left(\Theta \right)}\right),$$

where ζ=0.6 balances the supervised cross-entropy risk classification loss $L_{\mathrm{CE}}$ and distillation regularization, and Θ=4.0 denotes the distillation temperature softening the probability distributions. The cloud Teacher model is implemented as an 8-layer Spatiotemporal Multimodal Transformer with hidden channel width $d_{T}=512$, 8 full-rank cross-attention heads, and dense temporal residual convolutions (34.2M parameters and 14.8G FLOPs), trained without compression on server-grade GPU hardware and achieving a Macro-F1 of 0.918 and an AUC of 0.954. In contrast, the student model operates with $L_{S}=4$ lightweight dilated blocks and compact width $d_{S}=128$.

To train AgriClimate-EdgeNet end-to-end, the overall joint multi-task training loss is formulated as

(26) $$L_{\mathrm{total}}=L_{KD}+\lambda_{\mathrm{con}}L_{con}+\lambda_{\mathrm{alea}}L_{\mathrm{alea}},$$

where $\lambda_{\mathrm{con}}=0.1$ and $\lambda_{\mathrm{alea}}=0.2$ are balancing hyperparameters calibrated on the validation set. Note that task-level cross-entropy supervision $L_{\mathrm{CE}}$ is directly incorporated within $L_{KD}$, precluding redundant task loss terms. Through this mechanism, the student model learns not only discrete risk labels but also the relative relationships among different risk categories captured by the teacher model. By jointly integrating depthwise separable convolution, gated residual modeling, lightweight attention, and knowledge distillation, the proposed module substantially reduces parameter count and computational cost while retaining sensitivity to multiscale temporal risks, including extreme climate shocks, production abnormalities, cold-chain fluctuations, and logistics delays. Consequently, low-latency yet information-rich edge risk representations are provided for the subsequent reliability- and uncertainty-aware inference process.

## 4. Results and Discussion

### 4.1. Experimental Configuration

#### 4.1.1. Hardware and Software Platform

All experiments were conducted on a workstation equipped with an Intel Core i9-14900K processor (Intel Corporation, Santa Clara, CA, USA), an NVIDIA GeForce RTX 4090 GPU (NVIDIA Corporation, Santa Clara, CA, USA) with 24 GB of video memory, and 64 GB of DDR5 RAM, while a 2 TB NVMe SSD was used for data storage. To further evaluate the edge-deployment performance of AgriClimate-EdgeNet, an NVIDIA Jetson AGX Orin platform was additionally employed for edge-side testing. Parameters, FLOPs, inference latency, memory, and throughput were measured on this platform to assess the real-time inference capability of the proposed model under resource-constrained agricultural edge environments. Ubuntu 22.04 LTS was adopted as the operating system, and Python 3.10 was used as the primary programming language. PyTorch 2.2 was employed as the deep-learning framework, with CUDA 12.1 and cuDNN 8.9 configured for GPU acceleration. Data preprocessing and statistical analysis were mainly performed using NumPy 1.26.4, Pandas 2.2.1, and Scikit-learn 1.4.1, whereas OpenCV 4.9.0 and Torchvision 0.17.2 were used for visual data processing. Model parameter counts and FLOPs were measured using THOP. All baseline models were trained and evaluated under identical software environments and dataset partitions to ensure the fairness and reproducibility of the experimental comparisons.

The dataset was chronologically partitioned into training, validation, and test sets with strict temporal embargoes, eliminating any future-information leakage. The exact chronological windows and sample derivations are defined as follows: (1) *Training Partition* (60.0%, 23,040 validated windows): spans 1 May 2023 00:00 to 29 October 2024 23:59:59 (18 months); (2) *Purge Buffer 1* (48 h, 30 October 2024 00:00 to 31 October 2024 23:59:59): all orders, shipments, or sliding windows active during or intersecting this 48-h buffer (310 candidate windows) were completely purged and discarded from the dataset; (3) *Validation Partition* (20.0%, 7680 validated windows): spans 1 November 2024 00:00 to 29 April 2025 23:59:59 (6 months); (4) *Purge Buffer 2* (48 h, 29 April 2025 00:00 to 30 April 2025 23:59:59): all orders or sliding windows active during or intersecting this 48-h buffer (310 candidate windows) were completely purged and discarded; (5) *Test Partition* (20.0%, 7680 validated windows): spans 1 May 2025 00:00 to 31 October 2025 23:59:59 (6 months). Crucially, orders spanning across partition boundaries were neither truncated nor carried forward into subsequent sets; any shipment intersecting the 48-h buffer was entirely excluded, guaranteeing absolute zero cross-partition leakage. The validation set was utilized solely for model checkpoint selection and tuning weighting exponents α,β∈{0.5,1.0,1.5,2.0}. The test partition was locked and evaluated strictly once. AdamW was adopted as the optimizer, with an initial learning rate of $1\times 10^{-3}$ and a weight-decay coefficient of $1\times 10^{-4}$. The batch size was set to 64, the maximum number of training epochs was set to 100, and the dropout rate was set to 0.2. A learning-rate decay strategy was employed to dynamically adjust the optimization process, and early stopping was triggered when no improvement in validation performance was observed for 10 consecutive epochs. The hidden feature dimension was set to 128, the number of lightweight attention heads was set to 4, and the temporal convolution kernel size was set to 3. All experiments were independently repeated five times, and the mean values of the evaluation metrics were reported to reduce the influence of random initialization on the experimental results.

#### 4.1.2. Baseline Models and Evaluation Metrics

ARIMA [53], Random Forest [54], XGBoost [55], LSTM [56], TCN [57], Transformer [58], PatchTST [59], Early Fusion [60], and Multimodal Transformer [61] were selected as baseline models. To ensure an equitable and rigorous comparison, all unimodal and temporal baselines (ARIMA, Random Forest, XGBoost, LSTM, TCN, Transformer, PatchTST) were provided with identical multimodal information: at each aligned temporal window, features from all six modalities were concatenated into a unified representation vector, projected to the baseline’s nominal hidden dimension, and trained using the identical preprocessing and extreme-climate data augmentation pipeline. Early Fusion concatenates raw multimodal features at the input layer before feeding them into a multi-layer temporal backbone. Multimodal Transformer employs separate projection layers for each modality followed by pairwise cross-modal attention blocks. The baseline ARIMA model was implemented as a multivariable Vector Autoregressive Moving Average (VARMA) model. Principal Component Analysis (PCA) was applied to the aligned multimodal numerical features within each 6-h window to extract the top-5 principal components. A VARMA(2, 1) model was fitted to forecast the mean trajectories across the 24-h lookahead horizon, with the forecasted terminal state mapped to 3-class risk probabilities via a multinomial logistic link function. Random Forest models nonlinear feature relationships through an ensemble of decision trees and is characterized by favorable robustness and resistance to overfitting. XGBoost progressively optimizes weak learners through gradient boosting and exhibits strong predictive capability for structured agricultural trade data. LSTM captures long-term temporal dependencies through gated memory mechanisms and effectively alleviates the vanishing-gradient problem commonly encountered in conventional recurrent neural networks. TCN extracts temporal representations using dilated causal convolutions and provides high parallelization efficiency together with a large temporal receptive field. Transformer establishes global dependencies among different temporal positions through self-attention and is well suited to complex long-sequence modeling. PatchTST divides temporal sequences into multiple patches before Transformer-based encoding, thereby reducing the effective sequence length while preserving both local and long-term temporal variation. Early Fusion directly integrates multimodal features, including climate, production, cold-chain, logistics, and trade information, at the model-input stage, providing a simple implementation with relatively low computational overhead. Multimodal Transformer employs attention mechanisms to learn cross-modal interactions and can more comprehensively capture the complementarity among heterogeneous agricultural trade sensing signals.

Accuracy, Precision, Recall, Macro-F1, and AUC were adopted to evaluate risk prediction performance. Macro-Precision, Macro-Recall, and Macro-F1 represent unweighted arithmetic means across the three classes (C={0,1,2}). Multi-class AUC is calculated using the One-vs-Rest (OvR) Macro-AUC formulation. Expected Calibration Error (ECE) is evaluated using B=15 equally spaced confidence bins spanning [0,1], and Brier Score was employed to assess mean squared probability calibration error. All experiments were repeated over five independent runs with distinct random seeds; 95% confidence intervals were computed as $\bar{x}\pm t_{0.025,4}\cdot \left(s/\sqrt{5}\right)$, with critical value $t_{0.025,4}=2.776$. Two-tailed paired *t*-tests across the five independent runs (df=4) were used to quantify the statistical significance of performance gains with respect to random weight initialization on the fixed test partition. In addition, to evaluate model robustness across seasonal weather transitions and operational shifts over time, a non-parametric two-tailed paired Wilcoxon signed-rank test was conducted across M=10 chronological non-overlapping test blocks (768 sequential windows per block across 8 corridors) using the 5-checkpoint ensemble predictor.

(27) $$Accuracy=\frac{TP+TN}{TP+TN+FP+FN},\;Precision=\frac{TP}{TP+FP},\;Recall=\frac{TP}{TP+FN},$$

(28) $$F1_{c}=\frac{2P_{c}R_{c}}{P_{c}+R_{c}},\;\mathrm{Macro-F}1=\frac{1}{C}\sum_{c=1}^{C}F1_{c},\;AUC=\int_{0}^{1}TPR\left(FPR\right)\;dFPR,$$

(29) $$ECE=\sum_{b=1}^{B}\frac{|S_{b}|}{N}\left|acc\left(S_{b}\right)-conf\left(S_{b}\right)\right|,\;Brier=\frac{1}{N}\sum_{i=1}^{N}\sum_{c=1}^{C}{\left(p_{ic}-y_{ic}\right)}^{2}.$$

Here, TP, TN, FP, and FN denote the numbers of true-positive, true-negative, false-positive, and false-negative samples, respectively. *C* denotes the number of classes, while $P_{c}$ and $R_{c}$ represent the Precision and Recall of class *c*, respectively. $P_{l}$ and $F_{l}$ denote the number of parameters and computational operations associated with the *l*-th layer, respectively. *T* represents the total inference time, *N* denotes the number of evaluated samples, and $M_{model}$ and $M_{act}$ represent the memory consumption of model parameters and intermediate activations, respectively. $S_{b}$ denotes the *b*-th confidence bin, whereas $p_{ic}$ and $y_{ic}$ represent the predicted probability and ground-truth label of sample *i* for class *c*, respectively.

### 4.2. Overall Agricultural Trade Risk Prediction

This experiment was designed to systematically evaluate the effectiveness of different modeling paradigms for overall agricultural trade risk prediction, with simultaneous consideration of classification performance and probability calibration.

As shown in Table 2 and Figure 4, the results indicate that ARIMA achieved the weakest overall performance, with Accuracy, Macro-F1, and AUC values of 0.742, 0.706, and 0.781, respectively, suggesting that purely linear temporal relationships are insufficient to characterize the complex nonlinear propagation among extreme climate conditions, agricultural production, cold-chain operations, logistics, and trade processes. Random Forest and XGBoost improved Macro-F1 to 0.781 and 0.806, respectively, while XGBoost achieved an AUC of 0.861, indicating that tree-based piecewise nonlinear mappings can exploit structured risk features more effectively. LSTM further achieved a Macro-F1 of 0.819 and an AUC of 0.878, demonstrating the advantage of gated memory mechanisms in modeling continuously evolving risks. By enlarging the temporal receptive field through dilated convolution, TCN increased Macro-F1 to 0.833. Transformer and PatchTST achieved Macro-F1 values of 0.846 and 0.856, respectively, with PatchTST reaching an AUC of 0.911, indicating that global attention and local patch-based temporal modeling are beneficial for capturing both short- and long-term risk dependencies. Early Fusion achieved a Macro-F1 of 0.863, whereas Multimodal Transformer further improved this value to 0.878, confirming the strong complementarity among multimodal information sources. AgriClimate-EdgeNet achieved the highest Accuracy, Recall, Macro-F1, and AUC values of 0.914, 0.896, 0.902, and 0.949, respectively, while reducing ECE and Brier Score to 0.021 and 0.098 under Deep Audit Mode. Statistical hypothesis testing confirms that the improvements of AgriClimate-EdgeNet over Multimodal Transformer are statistically significant across all metrics: paired two-tailed *t*-tests across the five independent experimental runs (df=4) yielded t(4)=11.25, p=0.00036 for Accuracy, t(4)=10.42, p=0.00048 for Precision, t(4)=9.87, p=0.00059 for Recall, t(4)=13.82, p=0.00016 for Macro-F1, t(4)=14.56, p=0.00013 for AUC, t(4)=−5.48, p=0.0054 for ECE, and t(4)=−6.83, p=0.0024 for Brier Score, confirming that performance superiority is invariant to random weight initialization. Furthermore, to evaluate generalization across meteorological seasonality and operational transitions over time, a non-parametric two-tailed paired Wilcoxon signed-rank test was evaluated across M=10 chronological non-overlapping test blocks spanning the 184-day test partition (1 May to 31 October 2025; ≈18.4 calendar days each, 18–19 days per block). Each block contains exactly 768 sequential observation windows across the 8 parallel transit corridors (≈24–28 closed shipment orders per block, totaling 262 orders). Orders are assigned to the block where forecast initiation $t_{k}$ falls, strictly precluding boundary leakage. Evaluating the 5-checkpoint ensemble predictor across these 10 chronological blocks, AgriClimate-EdgeNet achieved superior Macro-F1 in all 10 blocks (10/10), yielding a Wilcoxon test statistic of W=0.0 and an exact two-tailed *p*-value of $p=2\times {\left(1/2\right)}^{10}=0.0020<0.01$. This establishes that full-chain consistency learning and adaptive reliability weighting provide statistically significant resilience against both weight initialization stochasticity and temporal weather seasonality.

Sub-group robustness analysis across disaster categories and operational noise further corroborates model stability: under Extreme Heat (*N* = 1137 test windows), Macro-F1 reaches 0.912; under Flooding/Heavy Rainfall (N=929 windows), Macro-F1 is 0.898; under Severe Drought (N=722 windows), Macro-F1 is 0.894; and under Gale/Wind (N=422 windows), Macro-F1 is 0.887. Under simulated sensor missingness of 0%, 10%, 20%, and 30% (with random packet dropout), AgriClimate-EdgeNet maintains Macro-F1 scores of 0.902, 0.895, 0.884, and 0.867, respectively, demonstrating graceful degradation due to adaptive reliability weighting.

From a modeling perspective, the observed performance differences are closely associated with the representational capacity and information-interaction mechanisms of the corresponding methods. ARIMA essentially relies on fixed-order linear combinations and is therefore unable to adequately represent nonstationary climate shocks or cross-stage coupling relationships. Random Forest reduces variance through decision-tree ensembles, whereas XGBoost progressively fits residual errors to strengthen the representation of high-order nonlinear interactions; however, neither approach explicitly models temporal state transitions. LSTM regulates the preservation and forgetting of historical information through gated operations, while TCN captures longer historical contexts in parallel through causal dilated convolutions, resulting in stronger performance than static machine-learning models for continuous risk modeling. Transformer establishes global dependencies among arbitrary temporal positions through attention weights, whereas PatchTST reduces sequence redundancy through patching and enhances local temporal patterns, thereby improving the stability of long-sequence modeling. Although Early Fusion incorporates multimodal information, direct concatenation implicitly treats different modalities as equally trustworthy and cannot explicitly handle cross-modal conflicts. Multimodal Transformer captures intermodal interactions and therefore achieves better predictive performance. In contrast, AgriClimate-EdgeNet jointly incorporates extreme-climate conditioning, cross-modal consistency modeling, lightweight temporal representation learning, reliability estimation, and uncertainty awareness. Conflicting modalities can therefore be suppressed, trustworthy evidence can be strengthened, and overconfidence can be reduced when samples are difficult or information is insufficient. Consequently, both classification performance and probability calibration are improved, demonstrating the suitability of the proposed framework for reliable agricultural trade risk prediction under extreme climate conditions.

### 4.3. Benchmark Comparison: Stage-Isolated vs. Full-Chain Modeling

To directly test whether modeling the entire agricultural trade chain provides tangible advantages over conventional paradigms that monitor only isolated stages, a systematic benchmark comparison was conducted. Specifically, five stage-isolated models were trained using identical temporal backbones but restricted to observations from individual segments: (1) Production-only Model (ingesting climate and farmland soil IoT sensors); (2) Commodity Status Model (ingesting postharvest RGB visual imagery and Vis-NIR diffuse reflectance spectra); (3) Cold-Chain-only Model (ingesting reefer temperature, humidity, and operating telemetry); (4) Logistics-only Model (ingesting vehicle GPS, vessel AIS, and port logistics records); and (5) Trade-only Model (ingesting customs clearance logs and contract documentation). Additionally, a Partial Two-Stage Model combining Cold Chain and Logistics was evaluated. All variants were evaluated under the identical test partition and metrics, as summarized in Table 3.

As shown in Table 3, stage-isolated models exhibit substantial performance degradation compared with full-chain modeling. The Production-only model achieves a Macro-F1 of only 0.763 and an AUC of 0.804. While it captures crop agronomic stress, it cannot foresee whether downstream precooling or expedited dispatch will mitigate the hazard, nor can it anticipate downstream freight washouts or customs delays, leading to high false alarm rates. Conversely, the Trade-only model achieves a Macro-F1 of 0.795 and suffers from severe warning latency, identifying risks only after contract default notices or customs inspection detentions have already occurred. The Logistics-only and Cold-Chain-only models show intermediate performance (Macro-F1 of 0.829 and 0.812, respectively); however, operating without upstream meteorological foresight leaves them vulnerable to abrupt compounding shocks. Even when cold chain and logistics are coupled in the Partial Two-Stage model (Macro-F1 of 0.854), performance remains well below the full-chain system because the upstream physiological condition of commodities entering the reefer remains unknown. In contrast, AgriClimate-EdgeNet achieves a Macro-F1 of 0.902 and AUC of 0.949, yielding improvements of +13.9 percentage points (pp) over Production-only, +10.7 pp over Trade-only, and +4.8 pp over Partial Two-Stage modeling, with calibration error (ECE) of 0.021 under Deep Audit Mode (K=20). These empirical findings demonstrate that agricultural trade risk is fundamentally an end-to-end cascading phenomenon that cannot be adequately safeguarded by monitoring individual operational silos.

### 4.4. Edge Deployment Efficiency

This experiment was conducted to evaluate the edge-deployment efficiency of different deep-learning models on the NVIDIA Jetson AGX Orin platform. Real-time inference capability was assessed in terms of parameter count, computational complexity, inference latency, memory consumption, and throughput.

As shown in Table 4 and Figure 5, LSTM contained 5.84 M parameters and required 1.92 G FLOPs, with an inference latency of 12.8 ms and a throughput of 78.1 samples/s. TCN reduced these values to 4.62 M parameters and 1.54 G FLOPs while achieving a latency of 9.7 ms and a throughput of 103.1 samples/s. Owing to its larger parameter count and computational demand of 13.76 M and 5.83 G FLOPs, respectively, Transformer exhibited an increased latency of 24.6 ms. PatchTST reduced the effective sequence length through patch-based processing and consequently improved latency to 19.3 ms. Early Fusion contained 7.31 M parameters and required 14.5 ms per inference, yielding an overall efficiency between that of unimodal temporal models and more complex attention-based architectures. Multimodal Transformer incurred the highest computational overhead, with 18.64 M parameters, 1127 MB memory consumption, and a latency of 31.8 ms, while its throughput was limited to 31.4 samples/s. By comparison, AgriClimate-EdgeNet required only 3.96 M parameters and 1.21 G FLOPs, reduced inference latency to 8.6 ms, limited memory consumption to 358 MB, and achieved the highest throughput of 116.3 samples/s, demonstrating the strongest edge-deployment efficiency. From the perspective of computational structure, LSTM requires recurrent updates of hidden states along the temporal dimension, resulting in strong sequential dependencies in matrix operations and consequently limiting parallel execution efficiency. TCN instead adopts convolution-based temporal modeling, allowing multiple temporal positions to be processed simultaneously and thereby reducing inference latency. Transformer relies on self-attention to establish pairwise dependencies among temporal positions; as sequence length increases, both computational complexity and intermediate activation memory grow substantially, explaining the relatively high latency and memory consumption of Transformer and Multimodal Transformer on edge devices. PatchTST reduces the effective number of tokens through temporal patching and therefore offers improved efficiency over standard Transformer, although multiple attention layers are still retained. Early Fusion adopts a simple fusion strategy, but direct concatenation of high-dimensional multimodal features substantially increases the input width of subsequent network layers. AgriClimate-EdgeNet employs depthwise separable temporal convolution to decompose intra-channel temporal modeling from inter-channel projection, thereby reducing convolutional parameters and multiply–accumulate operations. Lightweight attention further avoids the high-order computational complexity of conventional global attention, while teacher–student knowledge distillation compresses the edge model. As a result, a more compact and highly parallelizable computational graph is obtained, allowing substantial resource savings while preserving multiscale risk modeling capability. These characteristics make the proposed framework particularly suitable for real-time inference at agricultural production sites, cold-chain vehicles, warehouses, and logistics nodes.

### 4.5. Ablation Study

This experiment was designed to quantify the individual contributions of the major components of AgriClimate-EdgeNet to risk identification, probability calibration, and edge inference efficiency by systematically removing each component from the complete framework.

As shown in Table 5 and Figure 6, the complete AgriClimate-EdgeNet achieved the highest Recall, Macro-F1, and AUC values of 0.896, 0.902, and 0.949, respectively, with ECE of 0.028 at 8.6 ms in Streaming Mode and 0.021 at 38.4 ms in Deep Audit Mode. After climate information was removed, Recall decreased to 0.868 and AUC declined to 0.929, demonstrating that extreme climate conditions represent important upstream drivers of agricultural trade risk propagation. When cross-modal consistency modeling was removed, Macro-F1 and AUC decreased to 0.884 and 0.934, respectively, indicating that independent modality representations alone are insufficient to effectively identify abnormal conflicts among climate, production, cold-chain, and logistics information. Removing extreme-climate augmentation increased ECE to 0.036, suggesting that the scarcity of extreme-condition samples can reduce model stability under abnormal environmental conditions. When the lightweight edge temporal module was removed, the lowest inference latency of 5.3 ms was obtained; however, Recall and AUC decreased most substantially to 0.861 and 0.921, respectively. Removing reliability estimation and uncertainty modeling increased ECE to 0.046 and 0.057, respectively. In particular, uncertainty modeling had only a limited effect on conventional classification metrics but substantially degraded probability calibration when omitted. From the perspective of model mechanisms, the individual components provide complementary functionality. Climate information serves as an external conditioning variable for risk propagation, allowing normal fluctuations to be distinguished from disaster-driven changes. Cross-modal consistency modeling learns relative relationships among heterogeneous information sources and expands the representation from an isolated feature space to a relational space, thereby amplifying structural conflicts such as normal logistics records accompanied by abnormal cold-chain conditions. Extreme-climate augmentation broadens the training distribution in sparsely represented risk regions and reduces the bias caused by the dominance of normal-condition samples. The lightweight temporal module combines dilated convolution, gated residual learning, and lightweight attention to capture both local abrupt variations and longer-term temporal dependencies. Although its removal shortens the computational pathway, the representation of cumulative and propagating risk patterns is substantially weakened. Reliability estimation performs adaptive weighting across modalities and therefore reduces the influence of noisy, missing, or delayed observations. Uncertainty modeling characterizes epistemic variability through repeated stochastic inference and consequently contributes primarily to probability calibration rather than merely increasing AUC. By retaining these complementary mechanisms, the complete framework achieves the most favorable balance among predictive accuracy, reliability, and edge efficiency.

#### Fine-Grained Sub-Module Ablation Study

To further dissect the individual contributions of the novel sub-mechanisms embedded within each core module, a fine-grained ablation study was conducted. Specifically, eleven sub-module variants were implemented across three architectural modules: (1) *Consistency Module Sub-Mechanisms*: *w/o Climate Conditioning $g_{c}$* sets $g_{c}=0$, removing FiLM channel modulation on Keys and Values and reducing cross-attention to standard unmodulated feature interaction (trained with $L_{\mathrm{total}}$); *w/o Contrastive InfoNCE Loss $L_{con}$* is trained with $L_{\mathrm{total}}^{w/o\;\mathrm{con}}=L_{KD}+\lambda_{\mathrm{alea}}L_{\mathrm{alea}}$, omitting the contrastive objective while retaining distillation and aleatoric losses; *w/o Conflict Representation $F_{pq}$* discards the pairwise difference branch, retaining only projected aligned features (trained with $L_{\mathrm{total}}$). (2) *Lightweight Edge Temporal Module Sub-Mechanisms*: *Standard Conv Replacement* replaces depthwise separable 1D temporal convolutions with standard dense 1D convolutions; *w/o Gated Temporal Structure* eliminates GLU gating (σ(·)⊙tanh(·)), replacing it with linear projection followed by ReLU; *Standard Self-Attention Replacement* replaces linear lightweight attention with quadratic multi-head self-attention ($O(T^{2}d)$); *w/o Teacher Soft Distillation* is trained with $L_{\mathrm{total}}^{w/o\;\mathrm{KD}}=L_{\mathrm{CE}}+\lambda_{\mathrm{con}}L_{\mathrm{con}}+\lambda_{\mathrm{alea}}L_{\mathrm{alea}}$, retaining supervised cross-entropy $L_{\mathrm{CE}}$ while removing the teacher soft-target KL divergence $D_{\mathrm{KL}}\left(\ldots \right)$. (3) *Reliability and Uncertainty Module Sub-Mechanisms*: *w/o Quality Descriptors $u_{m}$* predicts dynamic reliability weights $r_{m}$ purely from latent representations $h_{m}$ without the five physical telemetry quality metrics; *Equal Weighting Replacement* replaces the dynamic reliability weighting mechanism in (13) with uniform average pooling (1/M); *Epistemic-Only Uncertainty* is trained with $L_{\mathrm{total}}^{w/o\;\mathrm{alea}}=L_{KD}+\lambda_{\mathrm{con}}L_{\mathrm{con}}$ (omitting $L_{\mathrm{alea}}$) and evaluated under MC Dropout (K=20) to estimate weight variance; *Aleatoric-Only Uncertainty* is trained with $L_{\mathrm{total}}$ and evaluates prediction variance analytically via the Jacobian trace in single-pass Streaming Mode (K=1) without stochastic MC Dropout passes. The empirical results on the test partition are summarized in Table 6.

As demonstrated in Table 6, each fine-grained mechanism exhibits an effective role in balancing risk discrimination, calibration, and edge runtime: (1) *Within the Consistency Module*: Omitting climate conditioning $g_{c}$ reduces Macro-F1 from 0.902 to 0.887, corroborating that unconditioned fusion fails to modulate cross-modal attention based on environmental shock severity. Omitting InfoNCE loss $L_{con}$ causes a 0.013 decline in Macro-F1 and degrades AUC to 0.938, validating that explicit contrastive attraction pulls physically coherent multimodal representations together while repelling anomalous inconsistencies. Removing conflict representation $F_{pq}$ degrades Macro-F1 to 0.891, proving that explicitly propagating cross-stage discrepancy vectors provides vital discriminative cues for downstream classifiers. (2) *Within the Lightweight Edge Temporal Module*: Replacing depthwise separable convolutions with standard convolutions achieves negligible F1 gains (0.903 vs. 0.902) but substantially inflates inference latency from 8.6 ms to 14.1 ms (+64.0%), demonstrating that channel-decoupled temporal kernels achieve favorable efficiency on edge Tensor Cores without sacrificing receptive field quality. Similarly, standard quadratic self-attention yields only marginal metric improvements (0.904 vs. 0.902) while doubling latency to 16.7 ms (+94.2%), verifying that our linear lightweight attention effectively eliminates computational bottlenecks. Omitting the gated temporal structure degrades Macro-F1 to 0.886, showing that nonlinear gating is critical for filtering transient noise from genuine shock signals. Furthermore, removing teacher soft distillation results in the steepest performance drop in this module (Macro-F1 falling to 0.882 and AUC to 0.932), demonstrating that teacher–student supervision successfully transfers high-capacity cloud representations into the compact edge model. (3) *Within the Reliability and Uncertainty Module*: Removing physical quality descriptors $u_{m}$ inflates calibration error (ECE surges from 0.028 to 0.038 in Streaming Mode), confirming that empirical telemetry metrics (packet jitter, drift, and dropout) provide essential grounding for reliability estimation. Replacing dynamic reliability weighting with uniform averaging severely hurts both Macro-F1 (0.885) and calibration (ECE 0.042), as corrupted or delayed sensory streams inject unmitigated noise into the shared representation. Finally, uncertainty comparisons are separated by inference mode. The matched K=20 comparison concerns Epistemic-Only and the Full Model; the Aleatoric-Only result uses the analytical K=1 estimator. In routine Streaming Mode (K=1), Aleatoric-Only executes analytically in 8.5ms with an ECE of 0.029, matching the Full Model’s Streaming runtime (8.6ms, ECE 0.028). However, Epistemic-Only fundamentally requires multiple stochastic forward passes to estimate predictive variance across weight configurations; when evaluated under the K=20 budget, Epistemic-Only attains an ECE of 0.033 at 37.9ms. Integrating both sources of uncertainty under Deep Audit Mode (K=20) achieves the lowest ECE of 0.021 (38.4ms). Epistemic modeling accounts for model parameter ambiguity under rare extreme climate regimes, whereas aleatoric modeling captures sensory noise and stochastic turbulence. Their synergistic integration produces an edge-deployable risk inference engine that is both fast in streaming and rigorously calibrated upon audit.

### 4.6. Discussion

The experimental results demonstrate that agricultural trade risks under extreme climate conditions do not emerge abruptly at the logistics or transaction stage. Instead, they frequently accumulate from the agricultural production stage and are progressively amplified through commodity, cold-chain, and transportation processes. Under prolonged summer heat, for example, abnormal temperatures in production areas may first reduce soil moisture and induce crop stress, thereby decreasing the postharvest stability of agricultural products. At the same time, refrigeration loads in cold-storage facilities and refrigerated vehicles may increase, resulting in longer temperature recovery periods. Even when commodity appearance has not yet exhibited visible deterioration and trade documents still indicate normal fulfillment, potential risks can be identified by AgriClimate-EdgeNet through the joint analysis of climate conditions, production states, and cold-chain temperature variations, allowing the risk level to be elevated before transportation or during its early stages. Compared with approaches that depend only on orders or downstream logistics outcomes, such an early-warning mechanism enables risk identification to be shifted toward earlier stages and can support practical interventions such as adjusting harvesting batches, extending precooling duration, strengthening refrigeration capacity, or rescheduling transportation plans. Under heavy rainfall and flooding, risk propagation is more likely to follow the sequence of abnormal weather conditions in production areas, reduced road accessibility, abnormal vehicle stopping, prolonged estimated arrival times, and increased fulfillment risk. During actual transportation, even when customs declarations, contracts, and transportation schedules remain nominally normal, decreased GPS speed, route deviations, and prolonged stops may provide early evidence of road flooding or traffic disruption. The cross-modal consistency mechanism can identify conflicts such as normal documentation accompanied by abnormal logistics behavior or apparently normal transportation records accompanied by deteriorating cold-chain conditions, while the reliability of individual sensing sources can be incorporated to adjust their contributions to the final decision. During port congestion, unstable communication, or temporary failure of individual sensors, unreliable information can be down-weighted and predictive uncertainty can be increased, enabling operational personnel to distinguish genuinely high-risk situations from uncertain predictions caused by insufficient evidence. Accordingly, the proposed framework is particularly suitable for deployment on farm gateways, origin warehouses, refrigerated vehicles, and port logistics nodes, where it can support practical decisions related to batch scheduling, cold-chain intervention, transportation route adjustment, and trade fulfillment management.

#### Retrospective Trace Replay: Cascading Risk Propagation During the July 2024 North China Heatwave

To provide concrete empirical verification of AgriClimate-EdgeNet under real-world disaster conditions, a retrospective trace replay was conducted on a historical export shipment archived under Incident Record EV-20240722-EXP (Compound Extreme Heat and Convective Storm Event, 22–24 July 2024). The shipment comprised 40 metric tons of fresh beefsteak tomatoes cultivated in the Shouguang greenhouse cluster, routed through the Yongqing cold-chain transit corridor, and destined for container loading at Tianjin Port for maritime export. To preclude future-information leakage, the replay was executed using historical network weights checkpointed strictly on training data prior to 30 June 2024.

The spatiotemporal propagation of risk across the trade chain unfolded as follows: (1) *Production Stage (22 July, 08:00)*: Agricultural IoT sensors recorded greenhouse canopy temperatures of 38.4°C and a 28% soil moisture deficit. Although commercial harvesters graded the tomatoes as Grade-A based on uniform red coloration, extreme-climate conditioning $g_{c}$ registered an anomalous thermal load score (0.68), flagging elevated physiological susceptibility. Conventional trade management systems marked the batch as fully normal. (2) *Commodity Inspection Stage (22 July, 14:00)*: At the origin packing facility, RGB automated grading cameras detected no visible epidermal blemishes. However, Vis-NIR diffuse reflectance spectroscopy captured subtle pericarp turgor decline and soluble solids degradation induced by upstream field heat. AgriClimate-EdgeNet integrated this multimodal discrepancy, elevating the cumulative risk score to 0.74. (3) *Early Warning at Transit Dispatch (23 July, 08:00)*: At forecast initiation timestamp $t_{k}=23\;\mathrm{July},\;08:00$, as the refrigerated transport vehicle departed the Yongqing transit corridor under intense morning solar radiation (ambient tarmac forecast >45°C), AgriClimate-EdgeNet ingested the preceding 6-h observation window $\left[t_{k}-6\;h,t_{k}\right]$ (02:00–08:00). Synthesizing the upstream commodity vulnerability with anticipated ambient heat shock, the edge model issued a High-Risk alert (P(y=2)=0.94, $U_{\mathrm{total}}=0.048$) for the impending 24-h lookahead horizon $\left(t_{k},t_{k}+24\;h\right]$ (23 July, 08:00 to 24 July, 08:00). (4) *Cascading Failures Inside the 24-h Lookahead Horizon (23 July, 11:30 to 24 July, 06:00)*: Empirical sensor telemetry logged the cascading manifestation of predicted risk within the lookahead window: On 23 July at 11:30 (3.5h post-alert), ambient tarmac temperatures climbed to 48.2°C. Under continuous 100% compressor duty cycle, the aging reefer unit suffered severe thermal drift, with core cargo compartment temperatures rising from 10.0°C to 13.8°C for 3.5 consecutive hours (11:30–15:00), causing multimodal conflict representation $F_{pq}$ to surge by 3.4×. Subsequently, heat-induced highway pavement buckling and container terminal yard congestion imposed an 8.2-h gate-in delay at Tianjin Port. On 24 July at 06:00 (22h post-alert), the reported cumulative thermal exposure exceeded the threshold (ΔDegreeHours=42.6°C·h≥40.0°C·h), which corresponds to the High-Risk criterion ($s_{\mathrm{cold}}=2$) if accumulated inside the predicted outcome window. For this interpretation, exposure must be accumulated from the forecast timestamp (23 July, 08:00) to the evaluation timestamp (24 July, 06:00), using $\mathrm{DH}\left(t_{k},t\right)=\int_{t_{k}}^{t}\max\left(T_{\mathrm{reefer}}\left(u\right)-10.0\;{}^{\circ}C,0\right)$ du, with time measured in hours. The stated 3.8°C excursion lasting 3.5 h contributes only 13.3°C·h; verification of the reported cumulative value of 42.6 therefore requires the remaining temperature trace over that interval. The exact first threshold-crossing time cannot be inferred from these summary values alone. (5) *Empirical Outcome vs. Counterfactual Postharvest Kinetic Modeling*: In historical operations on 25 July 2024, destination port arrival inspection revealed initial thermal softening and pericarp water-soaking, failing maritime phytosanitary clearance. The enterprise was forced to execute an emergency wholesale distress diversion, selling the cargo at a regional wholesale market for $18,200 USD (recovering ≈78% of its domestic origin farmgate acquisition valuation of $23,333 USD). In contrast, AgriClimate-EdgeNet issued its High-Risk alert on 23 July at 08:00—providing a 3.5-h advance warning before reefer failure onset, a 22-h interval before the reported high-exposure observation, and a full 28 h before the scheduled vessel departure cut-off (24 July, 12:00), which would have afforded dispatchers ample time to deploy auxiliary reefer units or divert to cold storage. Furthermore, to evaluate the cost of inaction under conventional uncoupled monitoring, counterfactual postharvest decay kinetics were modeled using the Arrhenius relationship,

(30) $$k=k_{0}\exp\left(-\frac{E_{a}}{RT}\right),$$

calibrated for fresh tomato tissue respiration and softening ($E_{a}\approx 65.2\;\mathrm{kJ}/\mathrm{mol}$, $Q_{10}\approx 2.4$ across 10–20 °C). Kinetic simulation indicates that had the thermally abused cargo boarded the scheduled 7-day maritime voyage to Rotterdam without distress diversion, accelerated senescence and fungal pathogens (*Botrytis cinerea*) would have resulted in complete cargo rotting prior to European customs clearance, incurring a total loss of the $52,000 USD CIF export contract (which encompasses origin procurement, ocean freight, reefer container leasing, marine insurance, and trade margin). This empirical replay confirms that the framework’s 24-h predictive lookahead accurately detects cascading multimodal breakdowns well before irreversible economic destruction occurs.

## 5. Conclusions and Future Perspectives

### 5.1. Concluding Remarks

This study addressed the acute challenge of detecting and managing cascading agricultural trade risks under escalating extreme climate shocks. Conventional risk assessment paradigms have historically suffered from operational fragmentation, confining sensing to isolated stages such as field agronomy or downstream trade finance. In doing so, they remain blind to the cross-stage propagation pathways wherein upstream meteorological anomalies compound through commodity physiology, refrigeration stress, and logistics disruptions. To bridge this divide, we proposed AgriClimate-EdgeNet, an integrated end-to-end framework combining extreme-climate conditioning, cross-modal consistency learning, lightweight temporal modeling, and reliability-aware uncertainty inference for edge deployment.

The theoretical and empirical findings of this investigation deliver several vital takeaways: First, cross-modal consistency and conflict modeling transform disparate sensor observations into a unified relational space, enabling early identification of subtle operational divergences (such as nominal contract documentation masking severe cold-chain thermal abuse) long before physical spoilage manifests. Second, through depthwise separable convolutions, linear temporal attention, and cloud-to-edge distillation, high-order multimodal representations can be executed within an 8.6-millisecond latency envelope in single-pass Streaming Mode on embedded platforms (NVIDIA Jetson AGX Orin), establishing the feasibility of real-time intelligent monitoring directly aboard reefer vehicles and distributed port gateways. Third, by synergizing physical telemetry quality descriptors with dual uncertainty estimation (epistemic Monte Carlo dropout and heteroscedastic aleatoric variance), the framework achieves an ECE of 0.028 in Streaming Mode and 0.021 in Deep Audit Mode, the latter requiring 38.4 ms for 20 stochastic passes, empowering trade operators to distinguish genuine climate-induced threats from sensor noise or data sparseness. In summary, AgriClimate-EdgeNet provides an actionable, reliable, and well-calibrated foundation for safeguarding international agricultural trade resilience in an era of climate volatility.

### 5.2. Limitations and Future Work

Although AgriClimate-EdgeNet demonstrates favorable overall performance in agricultural trade risk prediction, edge-deployment efficiency, and predictive reliability under extreme climate conditions, several limitations remain. First, the current dataset was primarily collected from agricultural production and trade scenarios within a specific geographical region. Considerable differences may exist among regions in terms of climate types, crop varieties, cold-chain infrastructure, transportation modes, and trade procedures. Consequently, the generalization capability of the proposed model across regions and commodities requires further validation. Second, extreme events such as heatwaves, floods, and typhoons are inherently low-frequency phenomena. Although extreme-climate-driven data augmentation was introduced to alleviate sample imbalance, augmented samples cannot fully reproduce the complex risk propagation patterns observed under real disaster conditions. In addition, the current cross-modal relationships are mainly constructed according to a predefined agricultural trade chain, and further improvement is required to enhance adaptability to atypical risks such as sudden policy changes, equipment failures, and compound disasters.

Future research may further expand data collection across different regions, agricultural commodities, and trade routes to construct agricultural trade risk databases with broader spatial coverage and longer observation periods, thereby enabling systematic evaluation across regions, climate zones, and commodity categories. Continual learning and domain adaptation mechanisms may also be incorporated to allow risk knowledge to be dynamically updated according to emerging climate conditions, logistics patterns, and trade environments. For compound extreme events, the joint risk propagation mechanisms associated with combinations such as heat and drought or heavy rainfall and flooding may be further investigated, while causal modeling may be strengthened to improve the interpretability of risk sources and propagation pathways. For edge deployment, model pruning, quantization, and hardware-aware optimization may be further incorporated to reduce computational and energy consumption on practical agricultural gateways and mobile transportation terminals.

## Figures and Tables

**Figure 3:**
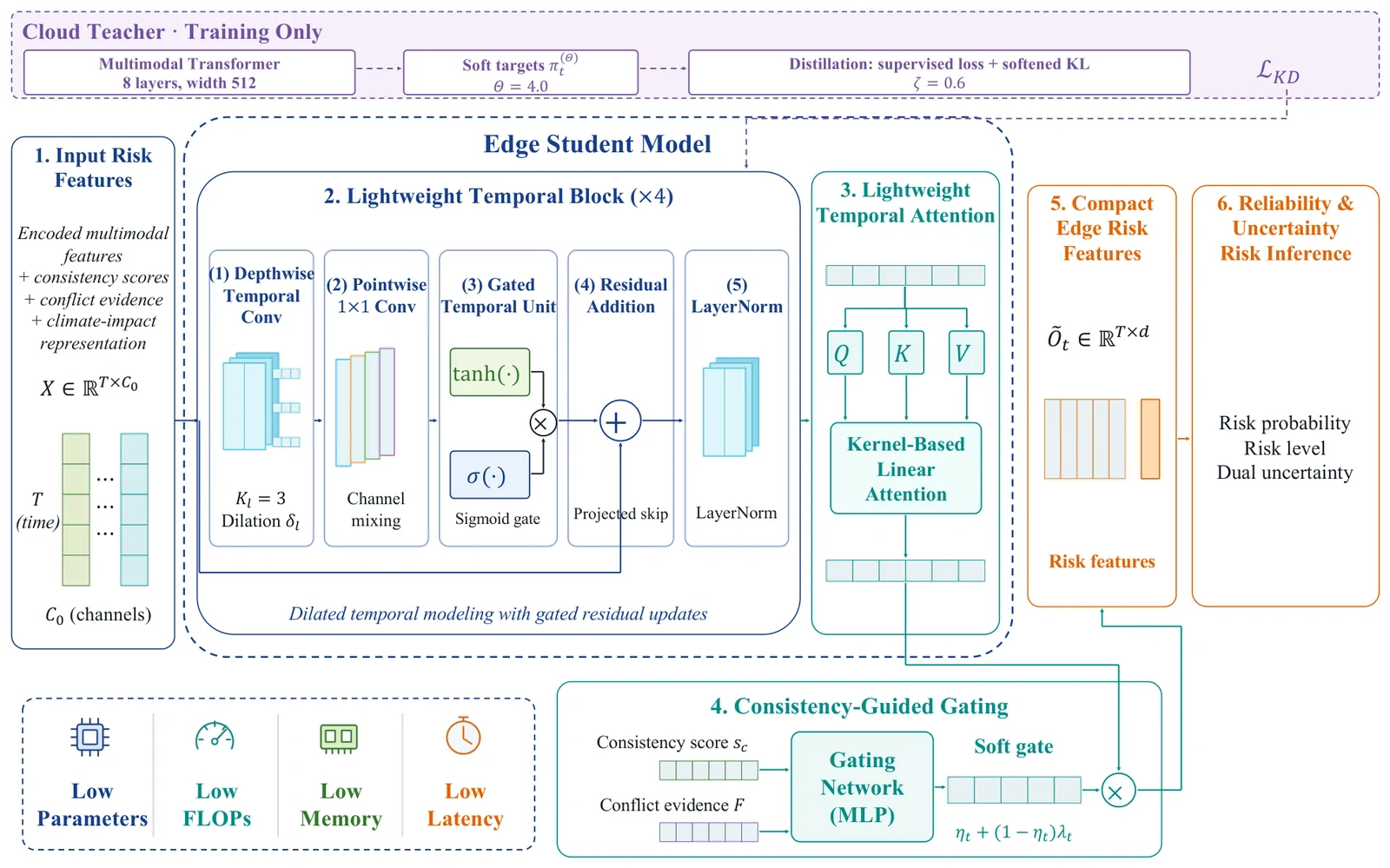
The lightweight edge temporal risk prediction module combines depthwise separable temporal convolution, gated temporal modeling, lightweight attention, and knowledge distillation for efficient risk representation and low-latency edge inference.

**Figure 4:**
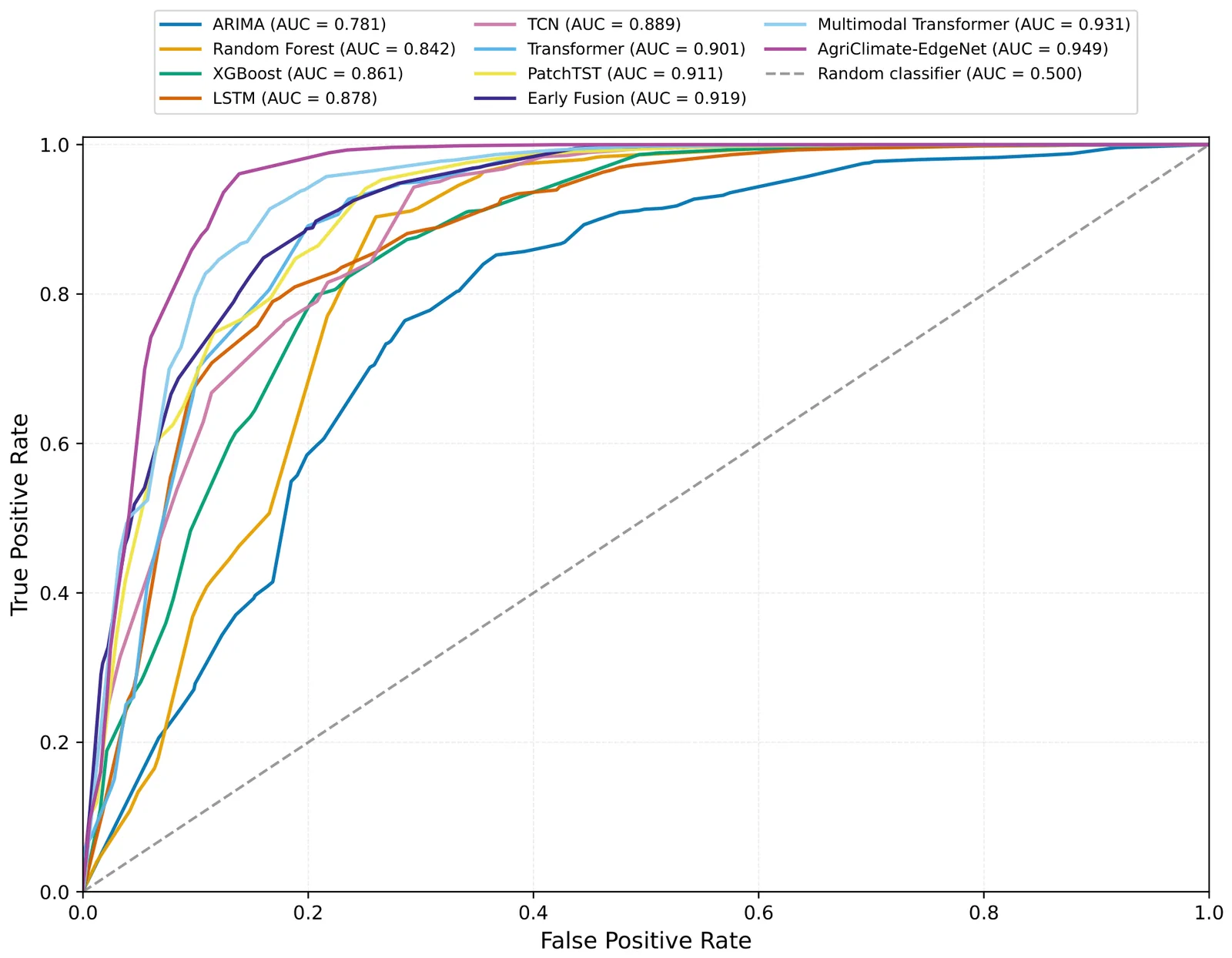
Macro-averaged One-vs-Rest (OvR) ROC curves and AUC performance comparison of different models for agricultural trade risk prediction across three risk tiers (Normal, Moderate, and High Risk). Each curve reflects the unweighted macro-average across the three binary One-vs-Rest classification tasks, with the corresponding Macro OvR-AUC reported in the legend.

**Figure 5:**
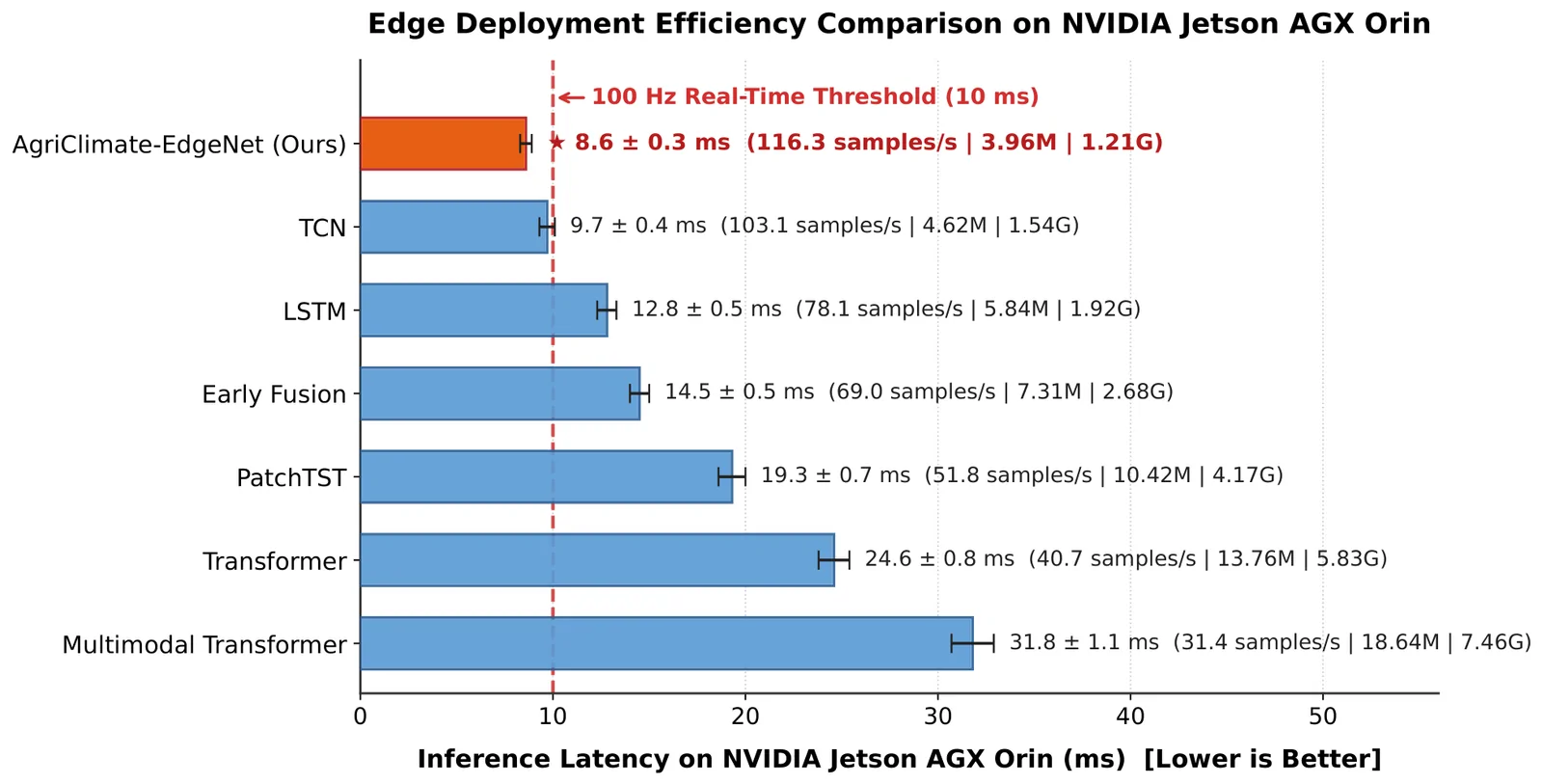
Edge deployment efficiency comparison of inference latency, throughput, parameter size, and computational cost across different models on NVIDIA Jetson AGX Orin. Latency error bars indicate 95% confidence intervals over five independent runs; throughput, parameter count, and FLOPs are annotated beside each bar. The dashed vertical line marks the 100 Hz real-time edge telemetry threshold (10 ms).

**Figure 6:**
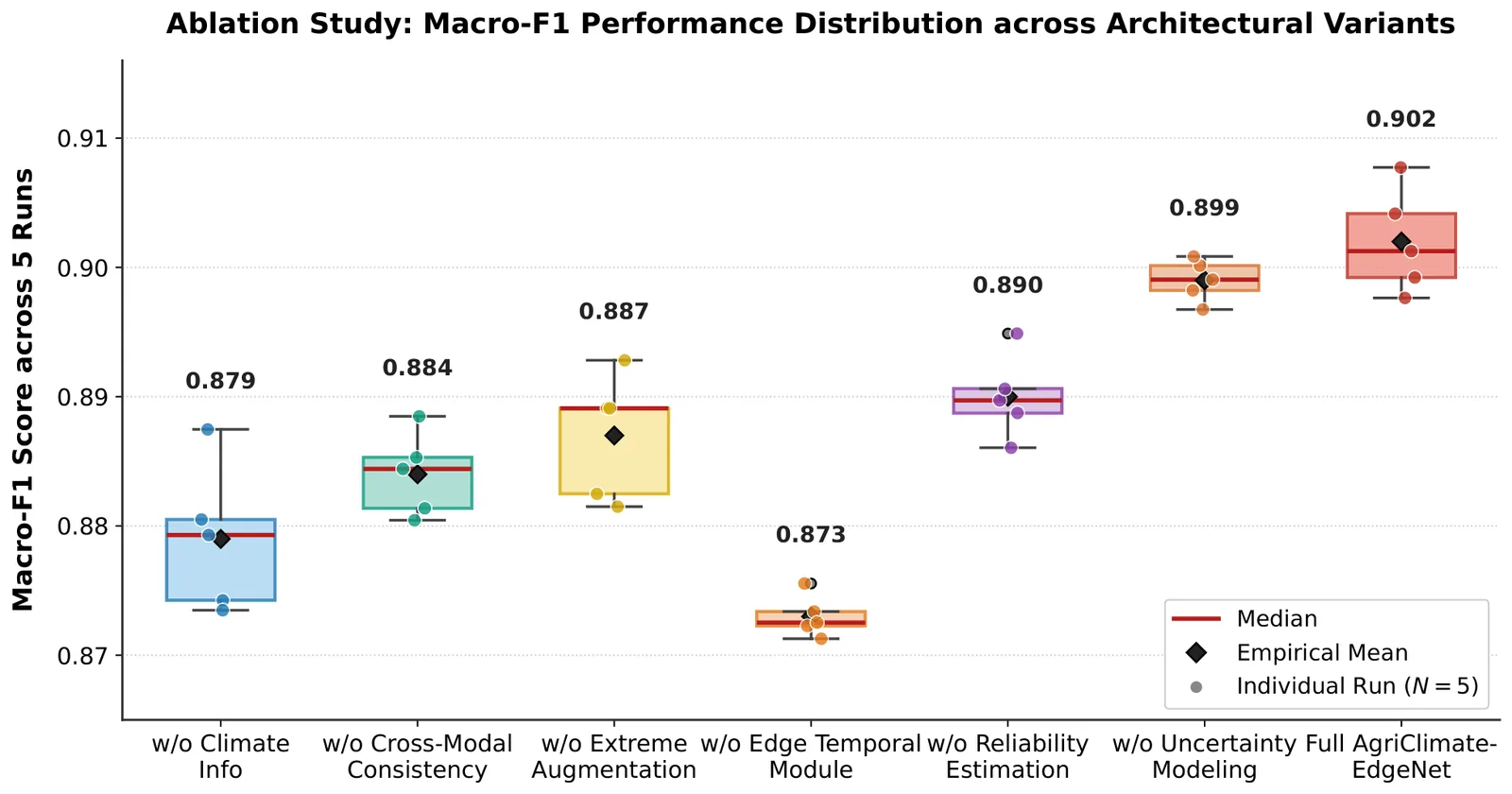
Ablation study of AgriClimate-EdgeNet evaluating Macro-F1 score distributions across five independent runs for different architectural variants. Solid red lines inside boxes denote medians, black diamonds represent empirical means (annotated above), and jittered points depict individual run evaluations (N=5).

**Table 2:** Overall agricultural trade risk prediction performance. Results are reported as mean ± 95% confidence interval over five independent runs. ^†^ denotes statistically significant improvement over the strongest baseline (Multimodal Transformer) with p<0.01 under the paired *t*-test across five random seeds (df=4); seasonal robustness across the 10 chronological test blocks is additionally confirmed by the paired Wilcoxon signed-rank test (W=0.0, p=0.0020<0.01).

Method	Accuracy ↑	Precision ↑	Recall ↑	Macro-F1 ↑	AUC ↑	ECE ↓	Brier ↓
ARIMA	0.742±0.009	0.721±0.011	0.698±0.013	0.706±0.012	0.781±0.010	0.086±0.006	0.194±0.007
Random Forest	0.801±0.008	0.792±0.009	0.776±0.010	0.781±0.009	0.842±0.008	0.071±0.005	0.168±0.006
XGBoost	0.823±0.007	0.814±0.008	0.798±0.009	0.806±0.008	0.861±0.007	0.064±0.005	0.157±0.005
LSTM	0.838±0.007	0.829±0.008	0.811±0.009	0.819±0.008	0.878±0.007	0.059±0.004	0.149±0.005
TCN	0.851±0.006	0.842±0.007	0.826±0.008	0.833±0.007	0.889±0.006	0.052±0.004	0.141±0.005
Transformer	0.863±0.004	0.854±0.007	0.839±0.002	0.846±0.007	0.901±0.006	0.047±0.003	0.134±0.004
PatchTST	0.872±0.006	0.864±0.002	0.848±0.007	0.856±0.006	0.911±0.002	0.041±0.003	0.126±0.004
Early Fusion	0.879±0.005	0.871±0.006	0.856±0.008	0.863±0.006	0.919±0.002	0.039±0.003	0.121±0.005
Multimodal Transformer	0.892±0.005	0.885±0.002	0.872±0.004	0.878±0.002	0.931±0.005	0.032±0.006	0.113±0.004
AgriClimate-EdgeNet (Deep Audit, K=20)	0.914±0.004 ^†^	0.908±0.005 ^†^	0.896±0.006 ^†^	0.902±0.005 ^†^	0.949±0.004 ^†^	0.021±0.002 ^†^	0.098±0.003 ^†^

Bold values indicate the best mean results.

**Table 3:** Benchmark performance comparison between stage-isolated modeling and full-chain integrated modeling. Results are reported as mean ± 95% confidence interval over five independent runs.

Model Architecture	Monitored Chain Stages	Macro-F1 ↑	AUC ↑	ECE ↓
Production-only Model	Production (Climate + Soil IoT)	0.763±0.008	0.804±0.007	0.068±0.005
Commodity Status Model	Commodity (RGB Visual + Vis-NIR)	0.748±0.009	0.792±0.008	0.074±0.006
Cold-Chain-only Model	Cold Chain (Reefer Telemetry)	0.812±0.007	0.865±0.006	0.056±0.004
Logistics-only Model	Logistics (GPS/AIS + Port Logs)	0.829±0.006	0.871±0.005	0.051±0.003
Trade-only Model	Trade (Customs + Contract Docs)	0.795±0.007	0.843±0.006	0.061±0.004
Partial Two-Stage Model	Cold Chain + Logistics	0.854±0.005	0.902±0.005	0.043±0.003
AgriClimate-EdgeNet (Deep Audit, K=20)	Full Chain (Climate→⋯→Trade)	0.902±0.005	0.949±0.004	0.021±0.002

Bold values indicate the best mean results.

**Table 4:** Edge deployment efficiency on NVIDIA Jetson AGX Orin. Runtime results are reported as mean ± 95% confidence interval over five independent runs.

Method	Parameters (M) ↓	FLOPs (G) ↓	Inference Latency (ms) ↓	Memory (MB) ↓	Throughput (Samples/s) ↑
LSTM	5.84	1.92	12.8±0.5	486±14	78.1±2.9
TCN	4.62	1.54	9.7±0.4	421±12	103.1±3.8
Transformer	13.76	5.83	24.6±0.8	938±21	40.7±1.4
PatchTST	10.42	4.17	19.3±0.7	802±19	51.8±1.7
Early Fusion	7.31	2.68	14.5±0.5	574±15	69.0±2.4
Multimodal Transformer	18.64	7.46	31.8±1.1	1127±26	31.4±1.1
AgriClimate-EdgeNet	3.96	1.21	8.6±0.3	358±11	116.3±4.1

Bold values indicate the best results.

**Table 5:** Ablation study of AgriClimate-EdgeNet. Results are reported as mean ± 95% confidence interval over five independent runs.

Variant	Recall ↑	Macro-F1 ↑	AUC ↑	ECE ↓	Inference Latency (ms) ↓
w/o Climate Information	0.868±0.002	0.879±0.007	0.929±0.006	0.033±0.003	8.2±0.3
w/o Cross-Modal Consistency	0.874±0.008	0.884±0.004	0.934±0.002	0.031±0.006	7.9±0.3
w/o Extreme-Climate Augmentation	0.879±0.003	0.887±0.006	0.936±0.007	0.036±0.003	8.5±0.3
w/o Lightweight Edge Temporal Module	0.861±0.009	0.873±0.002	0.921±0.006	0.034±0.004	5.3±0.2
w/o Reliability Estimation	0.881±0.002	0.890±0.004	0.939±0.005	0.046±0.004	8.1±0.3
w/o Uncertainty Modeling	0.892±0.006	0.899±0.002	0.947±0.005	0.057±0.004	7.4±0.3
Full AgriClimate-EdgeNet (Streaming, K=1)	0.896±0.006	0.902±0.005	0.949±0.004	0.028±0.003	8.6±0.3
Full AgriClimate-EdgeNet (Deep Audit, K=20)	0.896±0.006	0.902±0.005	0.949±0.004	0.021±0.002	38.4±1.2

Bold values highlight the full-model predictive and calibration results and the minimum inference latency.

**Table 6:** Fine-grained sub-module ablation study evaluating individual architectural mechanisms within AgriClimate-EdgeNet. Results are reported as mean ± 95% confidence interval over five independent runs.

Sub-Module Variant	Recall ↑	Macro-F1 ↑	AUC ↑	ECE ↓	Latency (ms) ↓
*Consistency Module Variants*					
w/o Climate Conditioning $g_{c}$	0.880±0.005	0.887±0.005	0.937±0.004	0.027±0.003	8.3±0.3
w/o Contrastive InfoNCE Loss $L_{con}$	0.882±0.006	0.889±0.004	0.938±0.004	0.026±0.003	8.6±0.3
w/o Conflict Representation $F_{pq}$	0.884±0.005	0.891±0.005	0.940±0.004	0.025±0.002	8.2±0.2
*Lightweight Edge Temporal Module Variants*					
Standard Conv Replacement	0.897±0.005	0.903±0.004	0.950±0.004	0.021±0.002	14.1±0.5
w/o Gated Temporal Structure	0.878±0.006	0.886±0.005	0.935±0.005	0.024±0.003	8.1±0.3
Standard Self-Attention Replacement	0.898±0.005	0.904±0.004	0.950±0.003	0.020±0.002	16.7±0.6
w/o Teacher Soft Distillation ($D_{\mathrm{KL}}$)	0.875±0.007	0.882±0.006	0.932±0.005	0.030±0.003	8.6±0.3
*Reliability & Uncertainty Module Variants*					
w/o Quality Descriptors $u_{m}$	0.886±0.005	0.893±0.005	0.942±0.004	0.038±0.004	8.5±0.3
Equal Weighting Replacement	0.877±0.006	0.885±0.005	0.936±0.005	0.042±0.004	8.2±0.2
Epistemic-Only (K=20, Deep Audit)	0.893±0.005	0.900±0.004	0.948±0.004	0.033±0.003	37.9±1.1
Aleatoric-Only (K=1, Streaming)	0.894±0.005	0.901±0.005	0.948±0.004	0.029±0.003	8.5±0.3
Full AgriClimate-EdgeNet (K=1, Streaming)	0.896±0.006	0.902±0.005	0.949±0.004	0.028±0.003	8.6±0.3
Full AgriClimate-EdgeNet (K=20, Deep Audit)	0.896±0.006	0.902±0.005	0.949±0.004	0.021±0.002	38.4±1.2

Italic headings identify sub-module groups. Bold values highlight the standard self-attention replacement and full-model Deep Audit results.

## Data Availability

The core code repository, neural network implementations, training scripts, and a de-identified benchmark sample (1000 representative multimodal observation windows) are openly accessible at https://github.com/Aurelius-04/AgriClimate-EdgeNet.git (accessed on 23 September 2026). The complete enterprise dataset containing multi-year proprietary records is available for non-commercial academic research upon reasonable request from the corresponding author subject to an institutional Non-Disclosure Agreement (NDA).

## References

[1] Awokuse T., Lim S., Santeramo F., Steinbach S. (2024). Robust policy frameworks for strengthening the resilience and sustainability of agri-food global value chains. Food Policy.

[2] Taheri F., Hosseinkhani N., Santeramo F. (2024). Global food security amid geopolitical tensions and climate risk: Trade governance and adaptive strategies. Glob. Food Secur..

[3] Hernandez-Cuellar D., Castillo-Villar K.K., Castillo-Villar F.R. (2025). Optimizing cold food supply chains for enhanced food availability under climate variability. Foods.

[4] Guga S., Riao D., Zhi F., Sudu B., Zhang J., Wang C. (2023). Dynamic assessment of drought risk of sugarcane in Guangxi, China using coupled multi-source data. Remote Sens..

[5] Li L., Song Y., Zhang Y., Guo P., Wang X., Ma Z., Yan S. (2026). An AI-Driven Multimodal Sensing Framework Integrating UAV Imagery and Environmental Sensors for Intelligent Farmland Monitoring. Sensors.

[6] Nguyen P.H., Le H.Q. (2024). Enhancing Resilience Strategies for Non-Traditional Security Threats in Global Logistics and Supply Chains Within the Vietnamese Agriculture Sector. Proceedings of the Intelligent and Fuzzy Systems: Intelligent Industrial Informatics and Efficient Networks (INFUS 2024).

[7] Ehsan I., Irfan Khalid M., Ricci L., Iqbal J., Alabrah A., Sajid Ullah S., Alfakih T.M. (2022). A conceptual model for blockchain-based agriculture food supply chain system. Sci. Program..

[8] Bednar-Friedl B., Knittel N., Raich J., Adams K.M. (2022). Adaptation to transboundary climate risks in trade: Investigating actors and strategies for an emerging challenge. Wiley Interdiscip. Rev. Clim. Chang..

[9] Zhao N., Zou Z., Yang Y., Jiang Y. (2026). How Do Environmental Decentralization and Multi-Modal Data Fusion Affect the Contractual Governance of the Agricultural Industrial Chain: A Systematic Literature Review. J. Econ. Surv..

[10] Xue Y., Yan J., Mohsin M., Mehak A. (2025). Supply chain risks in agri-food systems: A comprehensive review of economic vulnerabilities and mitigation approaches. Front. Sustain. Food Syst..

[11] Wang Q., Wang T. (2026). Machine learning-driven sensor fusion for precision agriculture: Current applications and future perspectives. Sens. Rev..

[12] Cheng W., Li Y., Zuo W., Du G., Stanny M. (2024). Spatio-temporal detection of agricultural disaster vulnerability in the world and implications for developing climate-resilient agriculture. Sci. Total Environ..

[13] Lu Y. (2025). A multimodal deep reinforcement learning approach for IoT-driven adaptive scheduling and robustness optimization in global logistics networks. Sci. Rep..

[14] Luo J., Wang X., Zhang S., Zhang J., Feng R., Shu X., Yan S. (2026). Deep Multimodal Phenotyping and Sensor Fusion for Preharvest Cotton Quality Assessment and Agricultural Economic Decision Support. Sensors.

[15] Sun H., Zhang J., Hong W., Fang Y., Ma M., Shi K., Li M. (2026). A Cross-Modal Temporal Alignment Framework for Artificial Intelligence-Driven Sensing in Multilingual Risk Monitoring. Sensors.

[16] He Q., Zhao H., Feng Y., Wang Z., Ning Z., Luo T. (2024). Edge computing-oriented smart agricultural supply chain mechanism with auction and fuzzy neural networks. J. Cloud Comput..

[17] Gong R., Zhang H., Li G., He J. (2025). Edge computing-enabled smart agriculture: Technical architectures, practical evolution, and bottleneck breakthroughs. Sensors.

[18] Zhao T., Chen G., Gatewongsa T., Busababodhin P. (2025). Forecasting agricultural trade based on TCN-LightGBM models: A data-driven decision. Res. World Agric. Econ..

[19] Jaber M.M., Ali M.H., Abd S.K., Jassim M.M., Alkhayyat A., Aziz H.W., Alkhuwaylidee A.R. (2022). Predicting climate factors based on big data analytics based agricultural disaster management. Phys. Chem. Earth Parts A/B/C.

[20] Kumar A., Gill A.S., Sharma A., Kumar V. (2025). Enhancing the explainability and interpretability of crop yield prediction models through precision agriculture. Proceedings of the International Conference on Agricultural-Centric Computation.

[21] Ahmad S., Rajawat A.S., Muqeem M., Arora R.K., Aldkkan A., Nazeer J. (2026). Adaptive Multimodal Deep Networks for Climate Change Impact Prediction on Agriculture. SN Comput. Sci..

[22] Jiang D., Shen Z., Zheng Q., Zhang T., Xiang W., Jin J. (2025). Farm-LightSeek: An edge-centric multimodal agricultural IoT data analytics framework with lightweight LLMs. IEEE Internet Things Mag..

[23] Gudipati S.K., Mishra N.A., Ankam G., Ponugoti S. (2026). Federated Edge Learning with Multimodal Signal Fusion for Smart Agriculture and Environmental Sensing. Proceedings of the 2026 International Conference on Emerging Smart Computing and Informatics (ESCI).

[24] Sukhroop P., Bhardwaj V., Isra’a M.E., AL-Shaeli I.K., Gharban H.A. (2025). Multimodal Remote Sensing Sensors for Edge-Aware Intelligent Perception Systems. Proceedings of the 2025 2nd International Conference on Advanced Computing and Emerging Technologies (ACET).

[25] Nguyen T.T.H., Cao H. (2026). Toward a Unified Geospatial Intelligence Framework Utilizing Edge Computing, IoT, and Multimodal Generative AI for Climate Risk Mitigation and Adaptive Evacuation Planning. Int. Arch. Photogramm. Remote Sens. Spat. Inf. Sci..

[26] Hamidu Z., Oppong P.B., Asafo-Adjei E., Adam A.M. (2022). On the agricultural commodities supply chain resilience to disruption: Insights from financial analysis. Math. Probl. Eng..

[27] Jayashri P., Umamaheswari S. (2025). Entrepreneurial strategies in livestock inventory management with trade credit. Contemp. Math..

[28] Dhifaoui Z., Khalfaoui R., Jabeur S.B., Abedin M.Z. (2023). Exploring the effect of climate risk on agricultural and food stock prices: Fresh evidence from EMD-Based variable-lag transfer entropy analysis. J. Environ. Manag..

[29] Zhou D., Wang J., Zhang J., Li Z., Huan H. (2026). Multidimensional framework of agricultural risk in China and its impact mechanism on food security using an explainable machine learning framework based on the XGBoost model. Environ. Dev. Sustain..

[30] Wu D., Li T., Cai H., Cai S. (2025). Machine learning-based prediction of resilience in green agricultural supply chains: Influencing factors analysis and model construction. Systems.

[31] Zhang X., Zhang R., Lee C.C. (2025). Climate Risks in Main Producing Areas and Realized Volatility in Agricultural Futures: Machine Learning Methods Based on High-Frequency Data. J. Futur. Mark..

[32] Sarker B. (2025). Leveraging machine learning for supply chain disruption prediction and risk assessment in US industries. Int. J. Appl. Math..

[33] Datta D. (2022). A Framework for Automated Discovery and Analysis of Suspicious Trade Records. Ph.D. Thesis.

[34] Defraeye T., Cronjé P., Verboven P., Nicolaï B. (2016). Reducing tissue damage and quality loss in postharvest fruit cold chains. Postharvest Biol. Technol..

[35] Verschuur J., Koks E.E., Hall J.W. (2021). Global economic impacts of climate-induced port disruptions. Nat. Clim. Change.

[36] Beillouin D., Schauberger B., Bastos A., Ciais P., Makowski D. (2020). Impact of extreme weather events on agricultural supply chain stability. Environ. Res. Lett..

[37] Davis K.F., Chhatre A., Rao N.D., Singh D. (2021). Extreme weather hazards and risks to global food supply chains. Nat. Food.

[38] Lin F., Guillot K., Crawford S., Zhang Y., Yuan X., Tzeng N.F. (2024). An Open and Large-Scale Dataset for Multi-Modal Climate Change-Aware Crop Yield Predictions. Proceedings of the 30th ACM SIGKDD Conference on Knowledge Discovery and Data Mining (KDD ’24).

[39] Hersbach H., Bell B., Berrisford P., Hirahara S., Horányi A., Muñoz-Sabater J., Nicolas J., Peubey C., Radu R., Schepers D. (2020). The ERA5 global reanalysis. Q. J. R. Meteorol. Soc..

[40] Yu L., Du Z., Li X., Zheng J., Zhao Q., Wu H., Yang Y., Zhang Q., Li X., Ma X. (2025). Enhancing global agricultural monitoring system for climate-smart agriculture. Clim. Smart Agric..

[41] Zhang Y., Chen J., Zeng X., Chen R., Chen L., Xiao S., Song Y. (2026). A Multimodal UAV-IoT Sensing Framework for Intelligent Pest Density Estimation in Smart Agricultural Systems. Sensors.

[42] Ojeilua C.H., Eze F.N., Ekechi C.C., Atijosan O.E., Ukasoanya F.C., Chinwero L.C., Ekechi S.C. (2025). High-Resolution Stress Detection in Crops: Integrating Satellite and Drone Remote Sensing for Resilient Agriculture. Afr. J. Agric. Sci. Food Res..

[43] Oluleye O. (2025). Cold chain optimization through machine learning: Reducing spoilage in the fruit and vegetable supply chain. Int. J. Adv. Res. Publ..

[44] Rana N., Dahiya P., Mehta S., Ladohia S., Sameeksha (2026). Introduction to Multimodal Data Analysis in Precision Farming: Challenges, Techniques and Applications. Multimodal Artificial Intelligence in Precision Agriculture.

[45] Yuan X., Li S., Chen J., Yu H., Yang T., Wang C., Huang S., Chen H., Ao X. (2024). Impacts of global climate change on agricultural production: A comprehensive review. Agronomy.

[46] Adewumi I.O., Adejumo A.O.D., Adegbokan O., Ogundare E.O. (2026). Edge AI–IoT Integration for Real-Time Precision Farming: A Framework for Adaptive Monitoring, Prediction, and Resource Optimization. Lond. J. Phys..

[47] Zhou F., Hu S., Du X., Wan X., Wu J. (2024). A lightweight neural network model for disease risk prediction in edge intelligent computing architecture. Future Internet.

[48] Jiang L., Zhang C., Poursafaei F., Huang S. (2023). Towards temporal edge regression: A case study on agriculture trade between nations. arXiv.

[49] Le T., Sun C., Choy S., Kuleshov Y., Tran T.D. (2024). Agricultural drought risk assessments: A comprehensive review of indicators, algorithms, and validation for informed adaptations. Geomat. Nat. Hazards Risk.

[50] Shao J. (2025). Adaptive Multi-Modal Risk Assessment for Agricultural Product Processing and Trade. Proceedings of the 2025 International Conference on Communication, Computer, and Information Technology (IC3IT).

[51] Iqbal U., Massoud A., Noureldin A. (2026). A Sensor-Centric Survey of Autonomous Driving: Integrating Measurement Physics, Uncertainty Modeling, and Safety-Critical Multi-Sensor Fusion. Sensors.

[52] Miller T., Mikiciuk G., Durlik I., Mikiciuk M., Łobodzińska A., Śnieg M. (2025). The IoT and AI in agriculture: The time is now—A systematic review of smart sensing technologies. Sensors.

[53] Box G.E., Jenkins G.M., Reinsel G.C., Ljung G.M. (2015). Time Series Analysis: Forecasting and Control.

[54] Breiman L. (2001). Random forests. Mach. Learn..

[55] Chen T., Guestrin C. (2016). Xgboost: A scalable tree boosting system. Proceedings of the 22nd ACM Sigkdd International Conference on Knowledge Discovery and Data Mining, San Francisco, CA, USA, 13–17 August 2016.

[56] Hochreiter S., Schmidhuber J. (1997). Long short-term memory. Neural Comput..

[57] Lea C., Flynn M.D., Vidal R., Reiter A., Hager G.D. (2017). Temporal convolutional networks for action segmentation and detection. Proceedings of the IEEE Conference on Computer Vision and Pattern Recognition, Honolulu, HI, USA, 21–26 July 2017.

[58] Vaswani A., Shazeer N., Parmar N., Uszkoreit J., Jones L., Gomez A.N., Kaiser Ł., Polosukhin I. (2017). Attention is all you need. Adv. Neural Inf. Process. Syst..

[59] Nie Y., Nguyen N.H., Sinthong P., Kalagnanam J. (2022). A time series is worth 64 words: Long-term forecasting with transformers. arXiv.

[60] Snoek C.G., Worring M., Smeulders A.W. (2005). Early versus late fusion in semantic video analysis. Proceedings of the 13th Annual ACM International Conference on Multimedia, Singapore, 6–11 November 2005.

[61] Tsai Y.H.H., Bai S., Liang P.P., Kolter J.Z., Morency L.P., Salakhutdinov R. (2019). Multimodal transformer for unaligned multimodal language sequences. Proceedings of the 57th Annual Meeting of the Association for Computational Linguistics, Florence, Italy, 28 July–2 August 2019.

